# Combined Oral and Topical Application of Pumpkin (*Cucurbita pepo* L.) Alleviates Contact Dermatitis Associated With Depression Through Downregulation Pro-Inflammatory Cytokines

**DOI:** 10.3389/fphar.2021.663417

**Published:** 2021-05-10

**Authors:** Maha Jameal Balgoon, Maryam H. Al-Zahrani, Soad Al Jaouni, Nasra Ayuob

**Affiliations:** ^1^ Department of Biochemistry, Faculty of Science, King Abdulaziz University, Jeddah, Saudi Arabia; ^2^ Department of Hematology/Pediatric Oncology and Yousef Abdullatif Jameel Chair of Prophetic Medical Applications (YAJCPMA), Faculty of Medicine, King Abdulaziz University, Jeddah, Saudi Arabia; ^3^ Department of Medical Histology, Faculty of Medicine, Damietta University, Damietta, Egypt

**Keywords:** skin, inflammation, CD4, CD68, COX2, iNOS, antioxidants, oleic acid

## Abstract

**Background:** Depression and contact dermatitis (CD) are considered relatively common health problems that are linked with psychological stress. The antioxidant, anti-inflammatory, and antidepressant activities of pumpkin were previously reported.

**Objectives:** This study aimed to evaluate the efficacy of the combined topical and oral application of pumpkin fruit (*Cucurbita pepo* L.) extract (PE) in relieving CD associated with chronic stress–induced depression and compare it to the topical pumpkin extract alone and to the standard treatment.

**Materials and Methods:** Forty male albino rats were exposed to chronic unpredictable mild stress (CUMS) for 4 weeks for induction of depression and then exposed to (1-fluoro-2, 4-dinitrofluorobenzene, DNFB) for 2 weeks for induction of CD. Those rats were assigned into 4 groups (*n* = 10 each); untreated, betamethasone-treated, PE-treated and pumpkin extract cream, and oral-treated groups. Treatments were continued for 2 weeks. All groups were compared to the negative control group (*n* = 10). Depression was behaviorally and biochemically confirmed. Serum and mRNA levels of pro-inflammatory cytokines, such as TNF-α, IL-6, COX-2, and iNOS, were assessed. Oxidant/antioxidant profile was assessed in the serum and skin. Histopathological and immunohistochemical assessments of affected skin samples were performed.

**Results:** Pumpkin extract, used in this study, included a large amount of oleic acid (about 56%). The combined topical and oral administration of PE significantly reduced inflammatory and oxidative changes induced by CD and depression compared to the CD standard treatment and to the topical PE alone. PE significantly alleviated CD signs and the histopathological score (*p* < 0.001) mostly through the downregulation of pro-inflammatory cytokines and the upregulation of antioxidants.

**Conclusion:** Pumpkin extract, applied topically and orally, could be an alternative and/or complementary approach for treating contact dermatitis associated with depression. Further studies on volunteer patients of contact dermatitis are recommended.

## Introduction

Depression is a common illness worldwide as more than 264 million people are suffering from it. Depressed persons suffer greatly and function poorly at work, at school, and in the family. There is a link between depression and physical health (WHO, 2020). The Lancet Psychiatry Commission concluded that patients with depression are at a higher risk for premature mortality and morbidity due to their unhealthy food choices, adverse effects of some offered treatments, and the effects of its symptoms (Firth et al., 2019).

The prevalence of contact dermatitis (CD, one of the skin inflammatory diseases, was described to be 10–40%, in general population; Ozgur et al. (2018). The occupational CD was reported to represent 70–90% of all occupational skin diseases and was described to deteriorate the functional capacity and the quality of life of the patient (Malik and English, 2015). Some studies have reported that psychological stress acts as a precipitating factor in triggering or worsening various skin diseases, including atopic contact dermatitis (Picardi et al., 2005; Amano et al., 2008). On the other hand, some biological and social studies support the causal effect of atopic CD on depression (Choi et al., 2018). CD was described to be linked to allergy and was proved to be associated with inflammatory factors (Thyssen et al., 2014). IL-6 and TNF-α have been reported to be elevated in mice showing behavioral despair and in patients with depression (Numakawa et al., 2014; Taraz et al., 2015). In a recent study conducted on depressed patients, the pro-inflammatory cytokine IL-6 was increased and TNF-α was correlated with psychological and cognitive fatigue (Pedraz-Petrozzi et al., 2020). Therefore, treatments with anti-inflammatory effect might be effective for treating CD and depression.

Recently, *Cucurbita* genus has received a great interest as it has been used in folk medicine in many countries for the treatment of gastrointestinal diseases and other clinical conditions (Salehi et al., 2019). Ethnopharmacological studies showed that pumpkin seeds were used to improve the erysipelas skin contamination (Yang et al., 2000). In addition, *Cucurbita pepo* L was used in some countries for treating burns and wounds (Ejaz et al., 2014), acne, dermatitis, and ecchymosis (Rigat et al., 2015). Traditional medicines, mainly Ayurvedic systems and Chinese medicine, have used different parts of the plant, including flesh of the fruits and seeds (Perez Gutierrez, 2016).

Pumpkin was described to have many health benefits, such as antioxidant, anti-inflammatory, and anti-fatigue (Wang et al., 2012; Nawirska-Olszańska et al., 2013). The effect of pumpkin on the central nervous system specifically was previously described, as the consumption of pumpkin seed oil (PSO) showed an efficacy in relieving the symptoms of ischemic stroke after ischemia-reperfusion (Shiri et al., 2016). In addition, pumpkin seed extracts were reported to produce antidepressant effects in rats comparable to that of imipramine, an antidepressant drug (George and Nazni, 2012). Sweetme Sweet Pumpkin (SPP) and *Cucurbita moschata* Duch. also showed an antidepressant effect in forced swimming test (FST)-induced depression comparable to that of fluoxetine, a classical antidepressant (Kim et al., 2016). They added that SPP and *Cucurbita moschata* Duch. increased brain tissue levels of the brain-derived neurotropic factor (BDNF), whereas they reduced the level of inflammatory cytokine (Kim et al., 2016). In a relatively recent review, pumpkin was described to have an antidepressant food score of 46%, indicating its antidepressant potential (LaChance and Ramsey, 2018). Recently, Dotto and Chacha (2020) endorsed conducting more animal and clinical trial–based researches in order to confirm the ameliorative effect of pumpkin seed on depression.

Regarding the effect of pumpkin on the skin, it was described that *Cucurbita pepo* L. seed oil even in small proportions significantly benefits the skin by increasing collagen synthesis and providing adequate photoprotection (Narendhirakannan and Hannah, 2013). Adding to that, *Cucurbita pepo* L. seeds’ oil has been described to be effective in enhancing healing of cutaneous diseases (Bardaa et al., 2016) and burns due to antioxidant and antibacterial activity (Bahramsoltani et al., 2017). Not only that, Bora et al., (2019) reported that the formulation including pumpkin seed oil and melatonin showed augmented anti-inflammatory effects occur in UV radiation–related sunburn along with the downregulation of inflammatory cytokines.

Putting together these studies, pumpkin fruit extract was hypothesized to improve both CD and depressive status due to its anti-inflammatory and antidepressive effects, respectively. This study aimed to evaluate the efficacy of the combined topical and oral application of *Cucurbita pepo* L. fruit extract in relieving CD associated with depression compared to topical pumpkin extract as well as the standard CD treatment.

## Materials and Methods

This study was approved by the Biomedical Research Ethics Committee at the Faculty of Medicine, King Abdulaziz University, Jeddah, KSA (reference number 45-20).

### Chemicals

Betamethasone valerate (BETA) was purchased from EPICO (19th of Ramadan City, Egypt) and used to treat the positive control group for pharmacological validation of the pumpkin extract (PE) cream. It was used at a dose of 75 μg (thinly and gently paint) using a specific brush twice a day for two weeks.

Fresh pumpkin (*Cucurbita pepo* L.) fruits were obtained from the local market at Jeddah, Saudi Arabia (voucher specimen: AQJ_123). It was identified in the King Abdulaziz University herbarium using specimens of herbarium, flora of KSA (Chaudhary, 2001). Voucher specimen was deposited in the herbarium, and the identification was verified by a botanist (Dr Faten Filimban, a certified plant taxonomist at Division of Botany, Department of Biology at King Abdulaziz University).

Extraction of pumpkin was done according to Wang et al. (2012) *.* First, the seeds were removed, and the raw fruits with skin were cut with a slicer, dried in a freeze dryer (FD5508; ILShinBase Co., Ltd., Korea), and crushed by grinding using an electrical machine. The obtained powder was passed through a 40-mesh sieve to get the fine powder to be stored in an airtight container.

The dried powder of pumpkin (50 g) was mixed with 450 ml of 80% ethanol at 37 C temperature for 1 day, left in shaker machine (JSSI-100T; JS Research Inc., Compact Shaking Incubator., Korea) for 1 day, and filtered with cotton and filter paper at the next day. This extraction process was repeated twice, and the excess solvent was evaporated under reduced pressure using a rotary vacuum evaporator (HS-2005S; HAHNSHIN Scientific Co., Ltd., Korea) to give an ethanol extract. It was left at fume hood for extra evaporation of ethanol, and then the extract was dried in freeze-dryer machine (FD5508; ILShinBase Co., Ltd., Korea). Pumpkin extract was stored in a suitable container till use after being diluted with hot distilled water in a dilution of 2:1 in an ultrasonication bath (Elmasonic S, lma Schmidbauer GmbHm Singen, Germany) and was administrated at a dose of 100 mg/kg by gavage once daily for two weeks (Wang et al., 2012)**.**

### Analysis of the PE

The chemical composition of PE was analyzed using trace gas chromatography GC-TSQ Evo 8000 mass spectrometer (Thermo Scientific, Austin, TX, United States) with a direct capillary column TG-5MS (30 m × 0.25 mm × 0.25 µm film thickness). The column oven temperature was initially held at 50°C, then increased by 5°C/min and held at 250 °C for 2 min, and increased to the final temperature of 300°C by 25°C/min and held for 2 min. The injector and MS transfer line temperatures were kept at 270 and 260°C, respectively; helium was used as a carrier gas at a constant flow rate of 1 ml/min. The solvent delay was 4 min, and diluted samples of 3 µL were injected automatically using an Autosampler AS1300 coupled with GC in the splitless mode in the PTV injector. EI mass spectra were collected at 70 eV ionization voltages over the range of m/z 50–650 in full scan mode. The ion source temperature was set at 250°C. The components were identified by comparison of their mass spectra with those of WILEY 09 and NIST 14 mass spectral database that is used in identification and study the chemical composition of unknown components in any extract (Wiley, 2006; Mikaia et al., 2014).

Analysis had been done in the qualitative type using Thermo Scientific™ Xcalibur™ 2.2 software, and all values were reported in relative percentage (Abd El-Kareem et al., 2016).

### Preparation of PE Cream

Simple ethanolic PE was formulated into cream as was previously described by Chen et al. (2016) with a modification. Oils used by Chen et al. were replaced by olive oil.

The PE cream was stored in a suitable container till the time of use at the dose (0.52 μL/mm^2^) reported by Bardaa et al. (2016), who used extracted oils of *Cucurbita pepo* L. for treating second-degree burns in rats.

### Experimental Design

Fifty male albino rats weighing 30–40 g were obtained from animal house of the King Fahd Medical Research Center (KFMRC) and left to acclimatize in the laboratory condition. Weights of the rats at the start of the experiment were ranged from 150 to 200 g. They were housed in plastic cages in an air-conditioned room at 22 ± 1°C and offered the standard animal chow and water *ad libitum*. Ten rats, which were left unexposed to neither stress nor CD, were assigned as the negative control group (control). The other forty rats were subjected to a CUMS procedure as they were exposed to different types of stressors at different times during the day for 4 weeks in order to prevent habituation to the stressors. The CUMS procedure was fully described in previous works (Ayuob et al., 2018).

### Induction of Skin Contact Dermatitis

A rectangular area (3 × 2 cm) on the dorsal surface of the rats was marked, and hair over this area was carefully shaved with an electrical shaving machine. Contact dermatitis was induced in the shaved area in all rats exposed to CUMS according to Simonetta and Bourgeois (2011). Briefly, rats were sensitized by painting 50 μL of 1-fluoro-2,4-dinitrofluorobenzene (DNFB) (0.1%, v/v) in acetone:olive oil 4:1 (AOO) onto the shaved dorsum of each animal for three consecutive days. Four days after sensitization, each rat was challenged by painting 30 μL of DNFB (0.2%, v/v) in AOO onto the dorsum every two days for 15 days. The painted areas were observed for signs of skin irritation for two weeks. These rats were then divided into 4 groups (*n* = 10 each). The positive control group was treated with the vehicle AOO. The CD + BETA group was topically treated with betamethasone cream, while the CD + PE group was topically treated with PE cream. CD + PE cream and the oral group were topically treated with PE cream and orally treated with PE. Topical treatments were performed using a special paint brush twice a day for two weeks.

### Behavioral Changes

In order to confirm the effect of CUMS, the forced swim test (FST) and elevated plus maize (EPM) were conducted for all rats after 4 weeks. Regarding FST, it was conducted according to Yankelevitch-Yahav et al. (2015). During this test, the rat was left to swim in a glassy cylindrical container with 15 cm depth of water at 25 ± 2 C. The rat was observed for 6 min by a technician blind to the experiment groups. The total time, in seconds, spent by the rat without mobility during the 6 min was determined. Immobility was defined as "the cessation of limb movement, except for the minor movement necessary to keep the rat afloat."

Regarding the elevated plus maze (EPM) test, it was performed according to Carobrez and Bertoglio (2005). The number of closed arm entries during 6 min and time spent by each mouse inside the open and closed arms were recorded in seconds.

### Serum Levels of Corticosterone and Pro-Inflammatory Cytokines

Blood samples were obtained for biochemical assessment from the intra-orbital sinus after completing the 4 weeks of exposure to CUMS and from the heart at the end of the experiment. Centrifugation was performed at 3,000 rpm for 15 min at 4 C to obtain the serum from the blood samples and was kept at −18°C. The corticosterone level was assessed to confirm induction of depression using enzyme-linked immunosorbent assay (ELISA) kits (ALPCO Diagnostics, Orangeburg, NY, United States) according to the manufacturer’s instructions.

Tumor necrosis factor-α (TNF-α) and interleukin-6 (IL-6) (Quantikine R&D system, United States) kits were measured in the serum using ELISA according to the manufacturer’s instruction. The optical density of each sample was determined in duplicate with a microplate ELISA reader set to 450 nm.

### Pro-Inflammatory Cytokines in the Skin

To assess the anti-inflammatory effect of treatment, the levels of cytokines such as TNF-α, IL-6, cyclooxygenase-2 (COX-2), and inducible nitric oxide synthase (iNOS) were measured in the skin. Samples of the affected skin were obtained and kept at −80°C for assessment of protein and gene expression. These frozen samples were homogenized and then centrifuged for 10 min at 5,000 g. The supernatant was used for ELISA (Thermo Fisher, Vienna, Austria) to assess the levels of cytokines.

### Oxidant/Antioxidant Profile in the Skin and Serum

Malonaldehyde (MDA) was used for the estimation of damage by reactive oxygen species (H_2_O_2_). The level of MDA was measured spectrophotometrically at 535 nm using the thiobarbituric acid reactive substance (TBARS) Assay Kit (Biodiagnostic; Egypt) according to the method described by Gamal et al. (2018).

In order to determine the superoxide dismutase (SOD) activity, nitroblue tetrazolium (NBT) was used. SOD Assay Kit (Biodiagnostic; Egypt) allowed very convenient SOD assaying through reduction of NBT to insoluble blue formazan. The method described by Packer (2002) was used.

Glutathione peroxidase (GPX) Kit (Randox Labs, Crumlin, United Kingdom) was used to assess GPX. To quantify catalase (CAT) activity, a calibration curve was generated for the assay using kits (Biodiagnostic; Egypt). The method used was described by Gamal et al. (2018).

### Quantitative Real-Time Polymerase Reaction

Total RNA extraction was done from the tissue samples using the TriFast^™^ reagent (PeqLab, Germany, Cat No.: 30–2010) according to the provided manufacturer’s protocol. The concentration of the purified RNA was estimated by NanoDrop 2000c Spectrophotometer (Thermo Scientific, United States). The extracted RNA from each sample was reverse-transcribed using the SensiFAST™ cDNA Synthesis Kit for qRT-PCR (Bioline United States Inc., United States, Cat No.: BIO-65053), following the manufacturer’s instruction. The synthesized cDNA was stored at −80°C until utilization for qRT-PCR.

The qRT-PCR reactions were performed using the SensiFAST™ SYBR Lo-ROX Kit (Bioline United States Inc., United States, Cat No.: BIO-94002) on the Applied Biosystems 7500 real-time PCR detection system (Life technology, United States). Gene-specific primers for rat-GAPDH internal control, rat-TNF-α, rat-IL-6, rat-iNOS, and rat-COX2 were designed using *Primer3 software* (*v.0.4.0*), while their specificity was checked using NCBI/Primer-BLAST program. The primers were purchased from Willowfort™ (United Kingdom) and the forward and the reverse primer sequences for the studied genes are presented in Supplementary Material S1. The PCR mixture was prepared as follows: 10 µL SensiFAST™ SYBR Lo-ROX Mix, 0.8 µL forward primers, 0.8 µL reverse primer, 2 µL template cDNA, and 6.4 µL nuclease-free water. The reaction mix was transferred to thermal cycler that was previously programmed to an initial hold at 95°C for 2 min followed by 40 cycles of 95°C for 15 s and then 60°C for 30 s. A negative control reaction containing no template was run in each experiment.

Melting curve analysis was carried out to prove specificity of PCR products, and the Ct value for each reaction was obtained from amplification plots. The relative quantification for each gene expression in the tissue samples was calculated using the comparative threshold (ΔΔCt) method with the GAPDH as the internal control gene. For overall fold change, it was calculated and linearized by the 2^−ΔΔCt^ arithmetic formula.

### Histological Techniques

At the end of the experiment, rats were anesthetized with 4% isoflurane (SEDICO Pharmaceuticals Company, Cairo, Egypt) in 100% oxygen and then euthanized by cervical dislocation. The chest wall was opened, and blood was obtained rapidly from the heart. Skin samples (2 × 2 mm) were immediately and gently dissected out and fixed in 10% neutral buffered formalin to be further processed for obtaining paraffin blocks. Paraffin sections at 4-μm thickness were prepared and stained with hematoxylin and eosin (Hand E) and Masson’s trichrome stain.

Another set of paraffin sections, at same thickness, was immunohistochemically stained using the streptavidin–biotin–peroxidase technique. Anti-CD68 antibodies (Biocare Medical, Pachieco, United States, at a dilution 1/100), Anti CD4 (Biocare Medical, Pachieco, United States, at a dilution 1/100), and anti-COX-2 (Biocare Medical, Pachieco, United States, at a dilution 1/100) were utilized in this study. CD68 and CD4 were used for detection of macrophages and T lymphocytes, respectively. The primary antibody was omitted while the secondary antibody IgG was added during staining of some slides to act as negative control slides. The nuclei were counterstained with hematoxylin. Brown cytoplasmic staining was considered positive reaction in the three antibodies. Olympus Microscope BX-51 (Olympus, Germany) connected to a digital camera and a computer was used for photographing the histological sections. Image-Pro Plus Analysis Software (Media cybernetics, United States) was used for semi-quantitative analysis of antibody immunoreactivity. The area percentage of immunoexpression of CD68^−^, CD4, and COX-2, used as an indicator of the extension of the reaction, was assessed in 30 fields using a × 40 objective lens and × 10 ocular lens. Epidermal thickness was measured in at least five fields in each slide, and the mean was calculated for each animal.

The histopathological scores of contact dermatitis were assessed using the scoring system previously described by Wang et al. (2015). This scoring system includes two parameters: infiltration of inflammatory cells (Grade 0, no changes; Grade one, few infiltrations; Grade 2, moderate infiltrations; and Grade 3, extensive infiltrations) and cuticulate *epidermis* (Grade 0, no changes; Grade 1, minor keratinization in epidermal tissue; Grade 2, obvious epidermal keratinization; and Grade 3, severe keratinization in epidermal tissue).

### Statistical Analysis

Statistical Package of Social Science Program (SPSS, SPSS Inc., Chicago, Illinois, United States) version 20 was used to analyze the raw data. Results were presented in the form of mean ± standard deviation (SD). The F-test (one-way analysis of variance) was used to compare the studied groups followed by the *post hoc* Bonferroni test to compare each two groups and avoid repeated comparisons. Significance was considered when *p* < 0.05.

## Results

### Compounds Detected in PE Using GC-MS

The constituents of PE, used in this study, mainly include oleic acid (about 56%), palmitic acid (about 8.9%), linolenic acid (3.5%), and linoleic acid (2.8%) beside many other compounds (Table 1). Some compounds of PE with anti-inflammatory effects were detected in this study such as oleic acid, palmitic acid, linolenic acid, betulin, and linoleic acid, while others have antimicrobial and antibacterial effects as 10-octadecenoic acid. Some compounds with antihistaminic and anti-eczemic effects were also detected like linolenic acid and linoleic acid, besides those with antioxidant effects as palmitic acid.

**TABLE 1:** Component of *Cucurbita pepo* L. extract identified using gas chromatography and mass spectrometer (GC-MS) analysis.

SN	Name of the compound	Molecular formula	Molecular weights	Retention time (in min)	Relative percentage (%)	NIST match factor Mikaia et al. (2014)	Activity
1.	Hexadecanoic acid, methyl Ester (palmitic acid methyl ester)	C_17_H_34_O_2_	270	21.50	3.02	839	Anti-inflammatory action through inhibition of the cyclooxygenase II enzyme Hema et al. (2011). Antioxidant, hypocholesterolemic, lubricant, and antiandrogenic Duke (1992)
2.	Hexadecanoic acid (palmitic acid)	C_16_H_32_O_2_	256	22.61	8.90	821	Anti-inflammatory through inhibition of phospholipase A2 Aparna et al. (2012). Antioxidant, hypocholesterolemic, lubricant, and antiandrogenic Duke (1992)
3.	9-Octadecenoic ACID (Z)-	C_18_H_34_O_2_	256	22.82	4.40	894	—
4.	10-Octadecenoic acid, methyl ester	C_19_H_36_O_2_	296	24.13	4.82	804	Antibacterial, antifungal, and antioxidant Asghar and Choudahry (2011)
5.	Butyl 9,12,15-octadecatrienoate	C_22_H_38_O_2_	334	24.73	1.60	743	No activity reported
6.	9-Octadecenoic acid (oleic acid)	C_18_H_34_O_2_	282	25.14	56.59	901	Anti-inflammatory actions through peroxisome proliferator–activated receptor gamma (PPAR-γ) activation Song et al. (2019)
7.	9,12-Octadecadienoic acid (Z,Z) (linolenic acid)	C_18_H_32_O_2_	280	25.42	3.52	855	Anti-inflammatory, hypocholesterolemic, antiandrogenic, antihistaminic, and anti-eczemic Duke (1992)
8.	*cis*-13-Octadecenoic acid (linoleic acid)	C_18_H_34_O_2_	282	26.01	1.75	796	Anti-inflammatory, antiandrogenic, antileukotriene—D4, hypocholesterolemic, and flavor Duke (1992)
9.	9-Octadecenoic acid (Z)-, anhydride (oleic anhydride)	C_36_H_66_O_3_	546	28.98	3.60	800	Anti-inflammatory Song et al. (2019)
10.	Linoleic acid ethyl ester (ethyl linoleate)	C_20_H_36_O_2_	308	29.21	1.08	782	No activity reported
11.	Stigmast-5-en-3-ol	C_29_H_50_O	414	35.65	3.08	725	Decrease endothelial leukocyte and platelet adhesion Duke (1992), Kikuchi et al. (2015)
12.	Betulin	C_30_H_50_O_2_	442	40.49	2.35	644	Anti-inflammatory and antitumor effect Jonnalagadda et al. (2017), Kikuchi et al. (2015)

### Confirmation of Depressive Status

**FIGURE 1:**
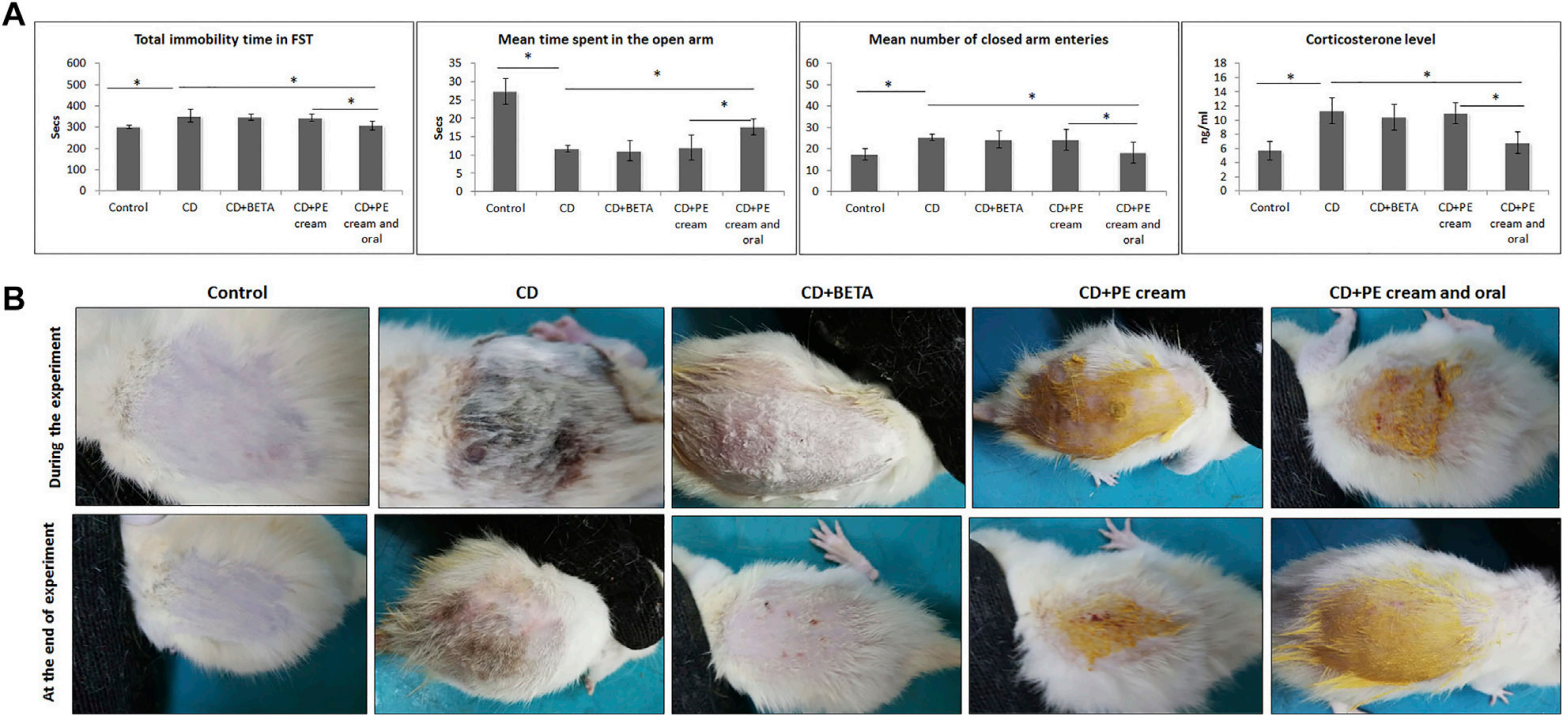
**(A)**: Confirmation of the depressive status in rats after exposure to CUMS through assessing of immobility time in FST, the EPM test, and the corticosterone level in the serum. **(B)**: Pumpkin extract affects the DNFB-induced dermatitis in rats morphologically as it improves the signs of contact dermatitis. CD: contact dermatitis, BETA: Betaderm, and PE: pumpkin extract. Data are presented as the mean ± SD, *n* = 10. Comparison between groups was done using the one-way ANOVA test followed by the Bonferroni *post hoc* test. FST: forced swimming test and EPM: elevated plus maize test.

After 4 weeks of exposing rats to CUMS, the development of depression-like behavior was confirmed through assessment of the behavioral changes and serum corticosterone level. A significant (*p* < 0.001) increase in the mean immobility time during FST was recorded in all CUMS-exposed rats compared to the control. Although no significant difference in the immobility time was recorded in either BETA-treated (*p* = 0.44) or PE cream–treated (*p* = 0.33) groups, a significant decrease (*p* < 0.001) was recorded in the group treated with topical and oral PE compared to the untreated and PE cream–treated groups (Figure 1A).

The EPM revealed a significant decrease (*p* < 0.001) in the time spent by CUMS-exposed rats in the open arm as well as a significant increase (*p* < 0.001) in the number of closed arm entries compared to the control.

Although no significant difference in both parameters was recorded in either BETA- or PE cream–treated groups, the group treated with topical and oral PE showed a significant increase (*p* < 0.001) in the time spent in the open arm as well as a significant decrease (*p* = 0.002, *p* = 0.01) in the number of closed arm entries compared to the untreated and PE cream–treated groups, respectively.

Administration of FLU and Pump significantly increased (*p* < 0.001) the time spent in the open arm compared to the CUMS group.

Also, the number of closed arm entries was significantly increased (*p* < 0.001) after CUMS exposure in comparison to the control group, while administration of FLU (*p* < 0.001) and Pump (*p* = 0.001) significantly decreased it in comparison to the CUMS group.

Regarding the serum corticosterone level, it showed a significant (*p* < 0.001) increase in the CUMS-exposed rats. Neither BETA nor PE cream significantly affected (*p* = 0.34, *p* = 0.78) the serum corticosterone level, respectively, while the group treated with topical and oral PE showed a significant reduction (*p* < 0.001) compared to the untreated as well as the PE-treated group (Figure 1B).

### Morphologic Appearance of Contact Dermatitis

Painting of the dorsal skin of rats with DNFB for two weeks resulted in the appearance of signs of CD that included hardness, dryness, and scaling. Application of BETA, and topical and oral PE for two weeks progressively improved these changes compared to the untreated group (Figure 1B).

### Anti-Inflammatory Effect of PE

**FIGURE 2:**
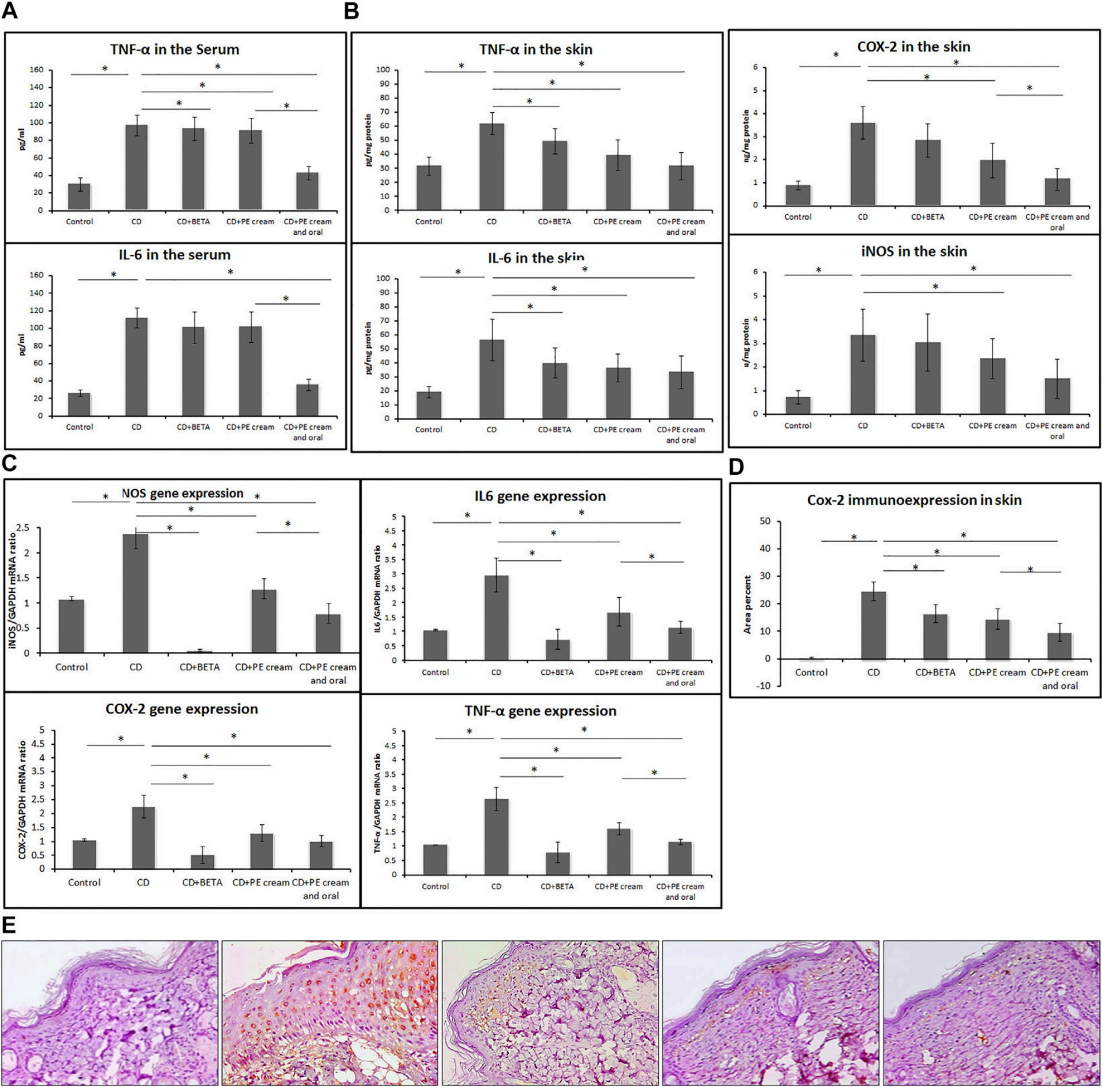
Pumpkin extract attenuates the pro-inflammatory cytokine secretion in DNFB-induced contact dermatitis. Levels of IL-6 and TNF-α in the serum **(A)** and in skin **(B)** were assessed using ELISA. The levels of mRNA of IL-6, iNOS, COX-2, and TNF-α **(C)** were assessed in the skin using qRT-PCR. Cox-2 immunoexpression **(D,E)** in the skin was assessed immunohistochemically. CD: contact dermatitis, BETA: Betaderm, and PE: pumpkin extract. Data are presented as the mean ± SD, *n* = 10. Comparison between groups was done using the one-way ANOVA test followed by the Bonferroni *post hoc* test.

To assess the anti-inflammatory effect of PE, pro-inflammatory cytokine levels were measured. It was found that serum TNF-α and IL-6 levels were significantly increased (*p* < 0.001) in the untreated CD group compared to the control, while their levels showed no significant difference in either BETA-treated (*p* = 0.44, *p* = 0.08) or PE-treated (*p* = 0.26, *p* = 0.06) groups. On the other hand, serum TNF-α and IL-6 significantly reduced (*p* < 0.001) in the group treated with topical and oral PE compared to both untreated CD and PE-treated groups (Figure 2A).

Levels of IL-6, iNOS, COX-2, and TNF-α showed a significant increase (*p* < 0.001) in the skin of the untreated CD group compared to the control, while they showed a significant reduction in the BETA-treated group (*p* < 0.001, *p* = 0.02, *p* = 0.03, *p* = 0.03), PE-treated group (*p* < 0.001, *p* = 0.03, *p* = 0.02, *p* < 0.001), and group treated with topical and oral PE (*p* < 0.001), respectively, compared to the untreated CD group (Figure 2B).

The mean expression of mRNA of IL-6, iNOS, COX-2, and TNF-α in the skin, assessed using qRT-PCR, was significantly upregulated (*p* < 0.001) in the untreated CD group, while it was downregulated in the BETA-treated group (*p* < 0.001), PE-treated group (*p* < 0.001), and group treated with topical and oral PE (*p* < 0.001) compared to the untreated CD group, respectively (Figure 2C).

Immunoexpression of COX-2 in the skin showed a significant upregulation (*p* < 0.001) in the untreated CD group, while it showed a significant downregulation (*p* < 0.001) in all treated groups compared to the untreated CD group. In addition, COX-2 immunoexpression was significantly downregulated (*p* = 0.001) in PE- and topical and oral PE-treated groups compared to the groups treated with PE cream only Figures 2D,E.

### Antioxidant Effect of PE

Contact dermatitis was associated with a significant increase (*p* < 0.001) in MDA in the skin as well as an insignificant (*p* = 0.06) increase in its level in the serum. Although topical treatment with DETA did not significantly reduce the MDA level in the skin, PE applied either topically (*p* = 0.01) or combined with oral PE (*p* < 0.001) could significantly reduce it compared to the untreated CD group. Regarding the MDA level in the serum, it was significantly reduced (*p* = 0.03) only in the topical and oral PE-treated group (Table 2).

**TABLE 2:** Effect of *Cucurbita pepo L.* on the oxidant/antioxidant profile in the serum and skin.

	Control	CD	CD + BETA	CD + PE cream	CD + PE cream and oral
MDA in skin (nm/mg protein)	1.66 ± 0.22	2.91 ± 0.68	2.53 ± 0.69	2.30 ± 0.51	1.79 ± 0.38
		P1 < 0.001	P2 = 0.12	P2 = 0.01	P2 < 0.001
				P3 = 0.34	P4 = 0.003
SOD in skin (µ/mg protein)	4.11 ± 1	1.74 ± 0.67	2.51 ± 1.2	2.65 ± 1.02	3.62 ± 1.35
		P1 < 0.001	P2 = 0.08	P2 = 0.04	P2<0.001
				P3 = 0.74	P4 = 0.03
GPX in skin (nmol/mg protein)	58.93 ± 5.2	35.7 ± 12.5	41.83 ± 9.2	46.02 ± 9.1	52.8 ± 9.2
		P1 < 0.001	P2 = 0.12	P2 = 0.01	P2 < 0.001
				P3 = 0.28	P3 = 0.09
CAT in skin (µ/mg protein)	116.7 ± 10.1	88.9 ± 17.6	97.4 ± 18.3	103.58 ± 13.8	111.4 ± 12.1
		P1 < 0.001	P2 = 0.18	P2 = 0.02	P2 = 0.001
				P3 = 0.32	P4 = 0.21
MDA in serum (nmoL/ml)	1.35 ± 0.14	1.73 ± 0.5	1.74 ± 0.34	1.64 ± 0.41	1.30 ± 0.19
		P1 = 0.06	P2 = 0.98	P2 = 0.65	P2 = 0.03 P4 = 0.09
				P3 = 0.63	
SOD in serum (µ/ml)	18.9 ± 2.9	9.9 ± 2.1	8.95 ± 2.6	9.10 ± 1.8	13.9 ± 3.6
		P1 < 0.001	P2 = 0.47	P2 = 0.33	P2 = 0.004
				P3 = 0.80	P4 < 0.001
GPX in serum (µ/ml)	58.6 ± 7.8	37.5 ± 4.9	35.16 ± 5.9	33.35 ± 6.6	47.9 ± 6.9
		P1 < 0.001	P2 = 0.47	P2 = 0.20	P2 = 0.003
				P3 = 0.57	P4 < 0.001
CAT in serum (µ/L)	0.41 ± 0.09	0.27 ± 0.08	0.23 ± 0.04	0.19 ± 0.06	0.37 ± 0.09
		P1 = 0.001	P2 = 0.39	P2 = 0.08	P2 = 0.02
				P3 = 0.36	P4 < 0.001

One-way analysis of variance (Kvetnansky et al., 2003) was used to compare the studied groups followed by *post hoc* test with the least significant difference. Results were presented in the form of mean ± standard deviation (SD). Significance was considered when *p* < 0.05.

P1, significance vs. the control.

P2, significance vs. the CD.

P3, significance vs. the CD + BETA.

P4, significance vs. the CD + PE cream.

Contact dermatitis was found to be accompanied with a significant reduction in SOD (*p* < 0.001), GPX (*p* < 0.001), and CAT (*p* < 0.001, *p* = 0.002) in the skin and serum, respectively, compared to the control. Although the DETA-treated group did not show a significant change in SOD, GPX, and CAT levels in either the skin or serum, the PE-treated group showed a significant increase (*p* = 0.04, *p* = 0.01, *p* = 0.02) in the skin but not in the serum. The group treated with topical and oral PE showed a significant increase in SOD (*p* < 0.001, *p* = 0.004), GPX (*p* < 0.001, *p* = 0.003), and CAT (*p* = 0.001, *p* = 0.02) levels in both the skin and serum, respectively (Table 2).

### Histopathological Alternations Associated With CD

**FIGURE 3:**
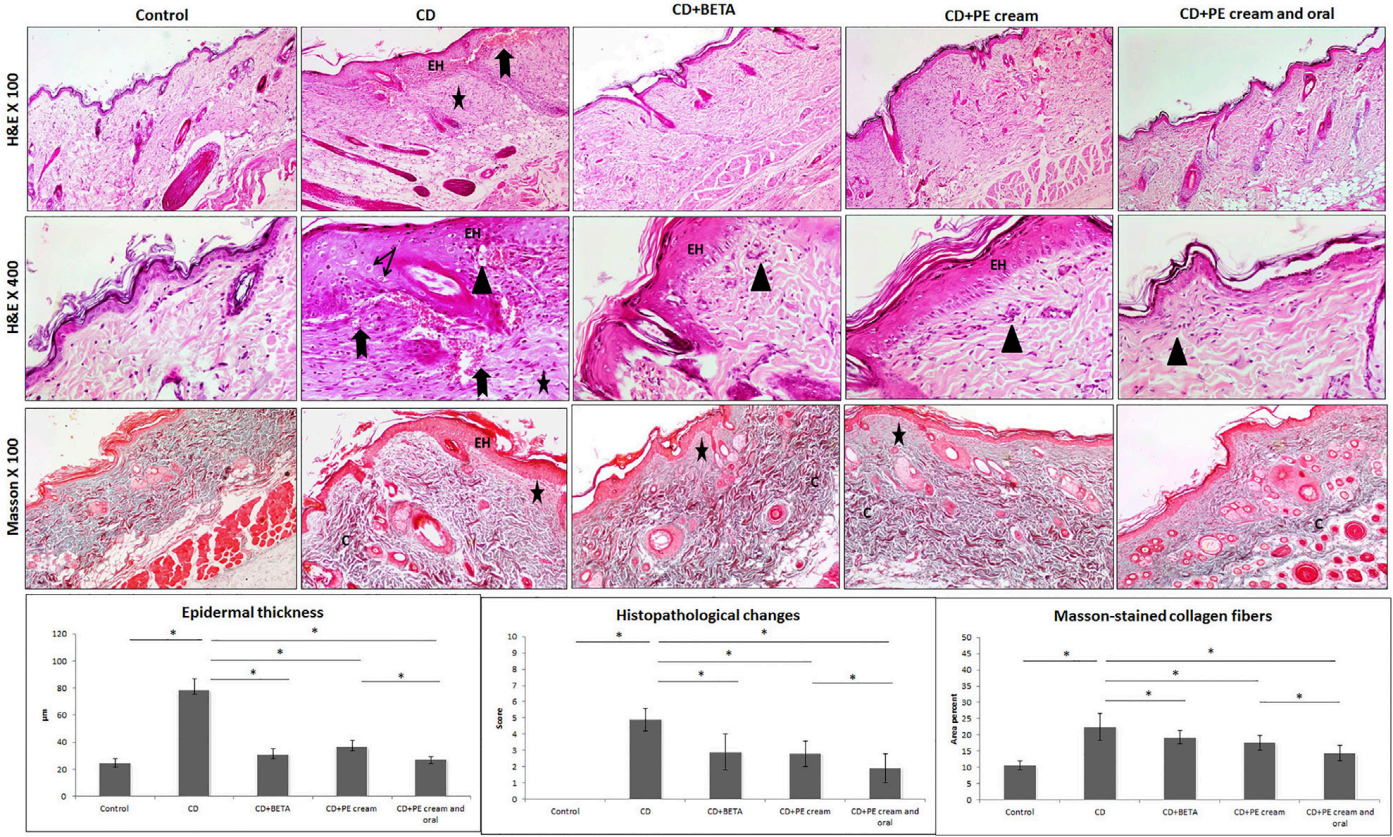
Pumpkin extract affects the DNFB-induced dermatitis in rats histologically. The histological changes in contact dermatitis (CD) group includes epidermal hyperplasia (EH), edema in superficial dermis (star), vacuolation of keratinocytes (arrow), inflammatory cell infiltrate (arrow head) and increased capillaries and hemorrhages (bifid arrow) as well as increased dense collagen fibers (C) in the dermis. The histological changes are improved in the treated groups (H&E and Masson stain). Data are presented as the mean ±SD, n= 10. Comparison between groups was done using One way ANOVA test followed by Bonferroni post *hoc* test

Histopathological assessment revealed an intact skin structure of the control group, while the CD group showed epidermal hyperplasia, vacuolation of keratinocytes, edema in the superficial dermis, inflammatory cell infiltrate, increased capillaries, and hemorrhages in some areas. A significant increase (*p* < 0.001) in the epidermal thickness, histopathological score of CD, and the area percent of Masson’s trichrome–stained dense collagen fibers was recorded in the CD group compared to the control (Figure 3).

Pumpkin extract administered as a topical cream alone or combined with oral PE markedly improved CD-associated histopathological changes. BETA-treated (*p* < 0.001), PE cream–treated (*p* < 0.001), and topical and oral PE-treated (*p* < 0.001) groups showed a significant decrease in epidermal thickness and the histopathological score of CD compared to the untreated CD group. A significant decrease in the area percent of Masson-stained dense collagen fibers was recorded in BETA-treated (*p* = 0.01), PE-treated (*p* < 0.001), and topical and oral PE-treated (*p* = 0.01) groups (Figure 3).

**FIGURE 4:**
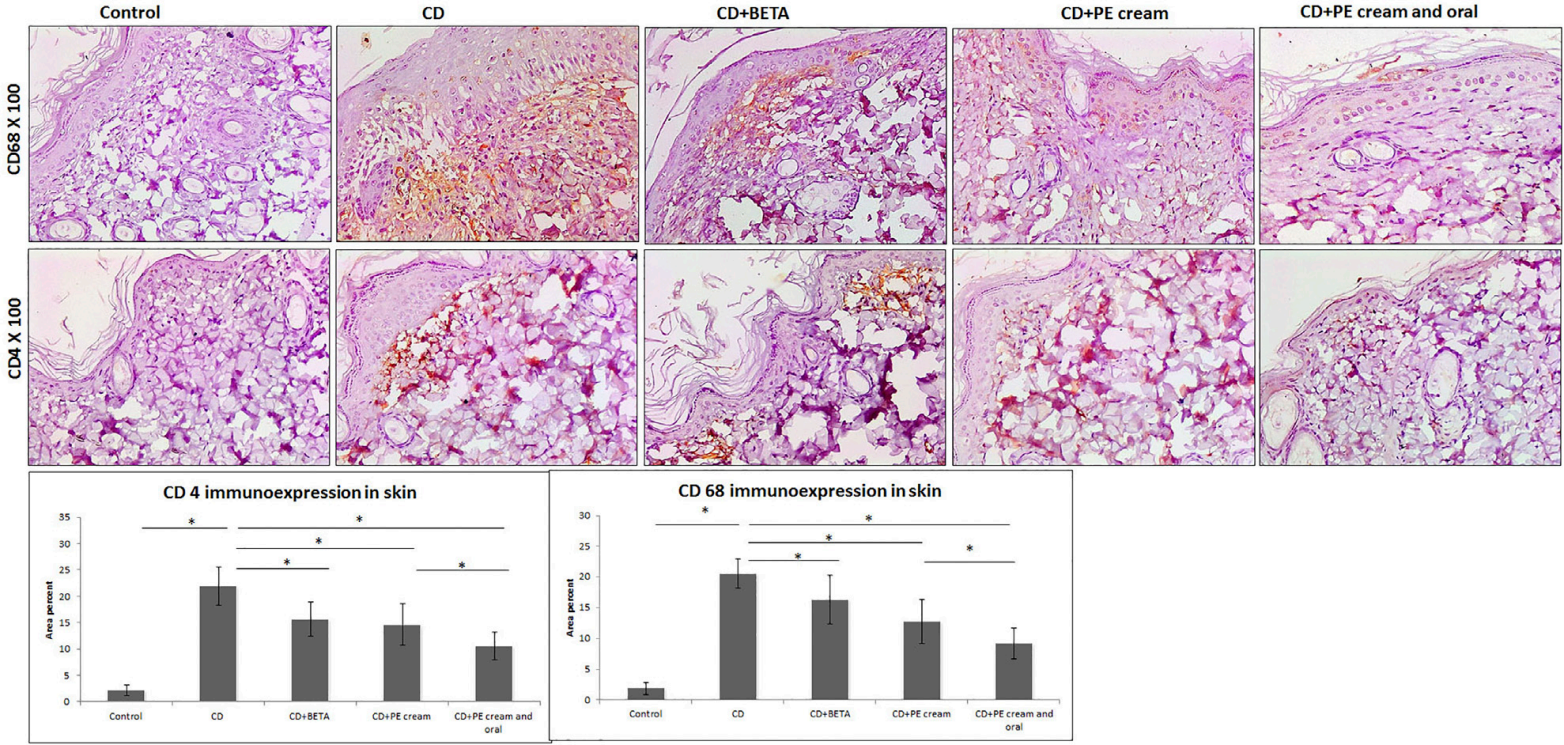
Pumpkin extract attenuates the inflammatory infiltrate in DNFB-induced contact dermatitis. CD68 and CD4 immunoexpression is downregulated as shown by immunohistochemistry. CD: contact dermatitis, BETA: Betaderm, and PE: pumpkin extract. Data are presented as the mean ± SD, *n* = 10. Comparison between groups was done using the one-way ANOVA test followed by the Bonferroni *post hoc* test.

Inflammatory cell infiltrate observed in the superficial dermis was specified and quantified immunohistochemically and found to include mainly CD68-positive macrophages and CD4-positive T lymphocytes. It was noticed that DNFB-induced CD associated with upregulation of CD68 and CD4 immunoexpression compared to the control. On the other hand, CD68 and CD4 immunoexpression was significantly downregulated in BETA-treated (*p* = 0.002, *p* < 0.001), PE-treated (*p* < 0.001), and topical and oral PE-treated groups, compared to the untreated CD group, respectively (Figure 4).

## Discussion

Stress influences the nature of some skin inflammatory diseases, such as psoriasis and allergic contact dermatitis (Buske-Kirschbaum et al., 2006). Stressful life situations were found to be related to the onset of symptoms in 16% of chronic urticaria cases (Malhotra and Mehta, 2008). This study was conducted to investigate the ability of combined topical and oral application of pumpkin fruit extract to biochemically and histopathologically improve CD in depressed rats.

The effect of chronic stress with the subsequent depression-like behavior on CD was not previously addressed in the experimental models. Among the well-established experimental models of human CD is DNFB-induced contact hypersensitivity in mice (Thomas et al., 1999). Chronic unpredictable mild stress (CUMS) procedure is considered a robust animal model for depression, which expressively contribute to the understanding of the mechanisms implicated in depression and the development of novel antidepressant drugs (Antoniuk et al., 2019). Therefore, these two models of depression and CD were adopted in this study. In the current study, exposure to CUMS successfully induced depression-like behavior as evidenced by the significant increase in the serum corticosterone level and prolongation in immobility time during the FST, and was confirmed by the EPM test. The latter was described as an important tool in the study of neurobiological signaling pathways involved in depression (Nestler et al., 2002). It is sensitive to all available antidepressant drugs commonly used to test the antidepressant effect of new drugs (Jeong et al., 2015).

iNOS, COX-2, and TNF-α are inflammatory mediators involved in the regulation of inflammatory reactions, including those of the skin, such as CD (Laouini et al., 2005; Orita et al., 2011; Danso et al., 2014). Both TNF-α and IL-6 were reported to be involved in the pathogenesis of depression (Taraz et al., 2015; Pedraz-Petrozzi et al., 2020). Hence, these cytokines were assessed, in this study, in order to investigate the anti-inflammatory effect of PE.

DNFB-induced CD was associated, in this study, with increased inflammatory cytokines TNF-α and IL-6 in the serum as well as IL-6, iNOS, COX-2, and TNF-α, at protein and mRNS levels, in the skin. These findings were in accordance with those reported by Kaur et al. (2014) in patients with acute and subacute allergic dermatitis as well as those reported by Qu et al. (2019) in rat model of contact dermatitis. Increased level of TNF- originated from the activated macrophages, T cells, and keratinocytes, and their release into the circulation in cutaneous inflammatory conditions was previously reported (Kerstan et al., 2011). In accordance with that, the number of macrophages, T cells, and keratinocytes was found to be increased in the dermis and epidermis of rats with CD, in this study. Although the inflammatory response plays a chief role in the protection of the host as well as in tissue repair, it can also damage the normal skin tissue (Cha et al., 2016). Based on that, inhibition of pro-inflammatory cytokine expression was described to improve dermatitis as it protects from extended adaptive immunity (Chang et al., 2018). Another reason for increased TNF-α and IL-6 in the serum in this study was the occurrence of CUMS-induced depression as they have been reported to be elevated in patients with depression and mice showing behavioral despair (Numakawa et al., 2014; Taraz et al., 2015).

In this study, topical application of PE extract to the areas affected with CD resulted in marked improvement of hardness, dryness, and scaling in a comparable degree to those animals treated with Betaderm cream, the standard treatment of CD. This morphological improvement was associated with a significant reduction in the inflammatory cytokines such as TNF-α and IL-6 in the serum as well as IL-6, iNOS, COX-2, and TNF-α in the skin. These findings were in agreement with those of Kim et al. (2016), who reported that SSP significantly reduced the protein levels of TNF-α and IL-6 in the serum of depressed animals. Regarding the effect on the skin, Bora et al. (2019) reported an enhancement in anti-inflammatory effects following the administration of PSO and melatonin formulation to UV radiation–induced sunburn evident by reduced inflammatory cytokines. Analysis of the PE compounds, conducted in this study, revealed the presence of many compounds with anti-inflammatory effect, for example, oleic acid, palmitic acid, and linolenic acid as well as some compounds with antihistaminic and anti-eczemic effects, for example linolenic acid and linoleic acid, besides those with antioxidant effect like palmitic acid. These compounds were behind the improving effect induced by PE in depressed rats with CD.

Disturbed oxidant/antioxidant profile manifested by increased MDA and decreased SOD, GPX, and CAT in the skin and serum was observed in DNFB-induced CD in this study. Similar findings were reported by Kaur et al. (2014) in patients with restricted allergic contact dermatitis. Disturbance in oxidant/antioxidant profile in CD was attributed to the consumption of radical-scavenging antioxidants as a result of increased free radical amounts (Serefhanoglu et al., 2009). The PE cream–treated group showed a significant improvement in antioxidant status in the skin, while the BETA-treated group did not show any improvement in this parameter. In addition, the group treated with topical and oral PE showed a significant improvement in the antioxidant profile in the skin and serum as well, and this might explain the marked improvement observed morphologically and histologically in this group.

The antioxidant properties of pumpkin fruit extract and seed oil were previously reported (Xia et al., 2003; Azizah et al., 2009; Bahramsoltani et al., 2017). It was said that natural products with antioxidant, anti-fatigue, and anti-inflammatory effects also exert an antidepressant-like effect (Jeong et al., 2015). All these previous activities were proved in pumpkin. Not only that, the antidepressant-like effect of pumpkin was previously reported by Kim et al. (2016), who found that oral SSP significantly reduced the immobility time in FST, increased the levels of brain-derived neurotropic factor (BDNF), and decreased the levels of IL-6 and TNF-α. Therefore, pumpkin was chosen, in this study, to relieve the combined manifestation of contact dermatitis with depression.

The morphological and histopathological alternations observed in CD in this study were in accordance with those observed by Zhou et al. (2016) in dinitrochlorobenzene-induced allergic CD in BALB/c mice (Alshathly and Alqahtani, 2017) in benzene-induced skin irritation in rats and (Qu et al., 2019) in oxazolone-induced CD in mice. Significant upregulation of Cox-2 immunoexpression in CD was among the findings recorded in this study and previous studies (Chang et al., 2018).

It was previously documented that T lymphocytes and macrophages have crucial roles in skin inflammatory diseases such as contact dermatitis and psoriasis (Qu et al., 2019); therefore, they were investigated in this study. Skin keratinocytes act as a potent source of pro-inflammatory cytokines and chemokines. Therefore, a particular dialog between keratinocytes and activated immune cells initiates and maintains the T cell–mediated immune responses in inflammatory lesions (Pivarcsi et al., 2005). T cells in the skin tissue were reported to sensitize and elicit a hypersensitive inflammation reaction (Pasparakis et al., 2014). These cellular events explained what was observed in the CD group in this study, which included keratinocyte hyperplasia, the overexpression of pro-inflammatory cytokines, the upregulated immunoexpression of CD4-positive T lymphocytes, and CD68-positive macrophages.

Pumpkin extract administered both topically and orally, in this study, markedly improved CD-associated histological changes in a comparable degree to that of BETA cream. CD68 and CD4 immunoexpression was significantly downregulated in BETA- and PE-treated groups, which implied reduced hypersensitivity reaction (Luo et al., 2014). No previous studies were found to describe the effect of pumpkin extract on CD associated with depression or chronic stress. The effect of caffeic acid (3,4-dihydroxycinnamic acid, CA), one of the six phenolic acids detected in pumpkin (*Cucurbita maxima*), on 12-O-tetradecanoyl-phorbol-13-acetate CD was previously studied (Zhang et al., 2014). They found that CA has anti-inflammatory activities in both acute and chronic contact dermatitis models through blocking of mRNA and protein synthesis of the cytokines, such as TNF-α, IL-6, and IL-1β, and neutrophil-mediated myeloperoxidase activity (Zhang et al., 2014).

Oleic and palmitic acids represent the main constituents of pumpkin fruit extract, utilized in this study. These findings are in partial agreement with those of Kim et al. (2012), Bardaa et al. (2016), and Bora et al. (2019). Both oleic and palmitic acids were reported to possess antioxidant, antidiabetic, and antiatherogenic effects (Cho et al., 2010). The link between depression and oleic acid was previously reported in the literature. Oleic, palmitic, and linoleic acids were described to be downregulated in depression (Conklin et al., 2010; Martín et al., 2010). Oleic acid in specific was reported to inhibit the production of Aβ peptide and amyloid plaque Alzheimer disease-type neuropathology both *in vitro* and *vivo* (Amtul et al., 2011). More recently, oleic acid–mediated neuroprotection might be attributable to its anti-inflammatory actions through peroxisome proliferator–activated receptor gamma (PPAR-γ) activation (Song et al., 2019). Regarding hexadecanoic acid (palmitic acid), it was described as an inhibitor of phospholipase A (2), and therefore considered as an anti-inflammatory compound (Aparna et al., 2012).

The improvement in CD as well as CUMS-induced depression, observed in the PE-treated group morphologically and behaviorally and were evident biochemically and histopathologically, is attributed mainly to the anti-inflammatory and antioxidant effects of pumpkin compounds that were detected in this study. Getie et al. (2002) reported that the antioxidant effect of any drug prevents cell damage, promotes DNA synthesis, increases vascularity, increases the strength of collagen fibers, and improves the viability of collagen fibrils.

In conclusion, topical application of pumpkin extract when combined with the oral administration was superior to the topical application alone in attenuating inflammation and oxidative changes induced by contact dermatitis associated with chronic stress–induced depression. These results imply that pumpkin can alleviate symptoms of contact dermatitis and depression through the downregulation of pro-inflammatory cytokines and enhancing the antioxidant status. Therefore, pumpkin extract, applied topically and orally, could be an alternative and/or complementary approach in contact dermatitis associated with depression-like behavior. Further studies to test this effect on volunteer patients of contact dermatitis are recommended.

## Data Availability

The original contributions presented in the study are included in the article/Supplementary Material; further inquiries can be directed to the corresponding author
