# Supplementary material for: Combined Oral and Topical Application of Pumpkin (Cucurbita pepo L.) Alleviates Contact Dermatitis Associated With Depression Through Downregulation Pro-Inflammatory Cytokines
Source: Front Pharmacol. 2021 May 10;12:663417. doi: 10.3389/fphar.2021.663417 (PMC8141732; doi:10.3389/fphar.2021.663417)

My GC-MS Report

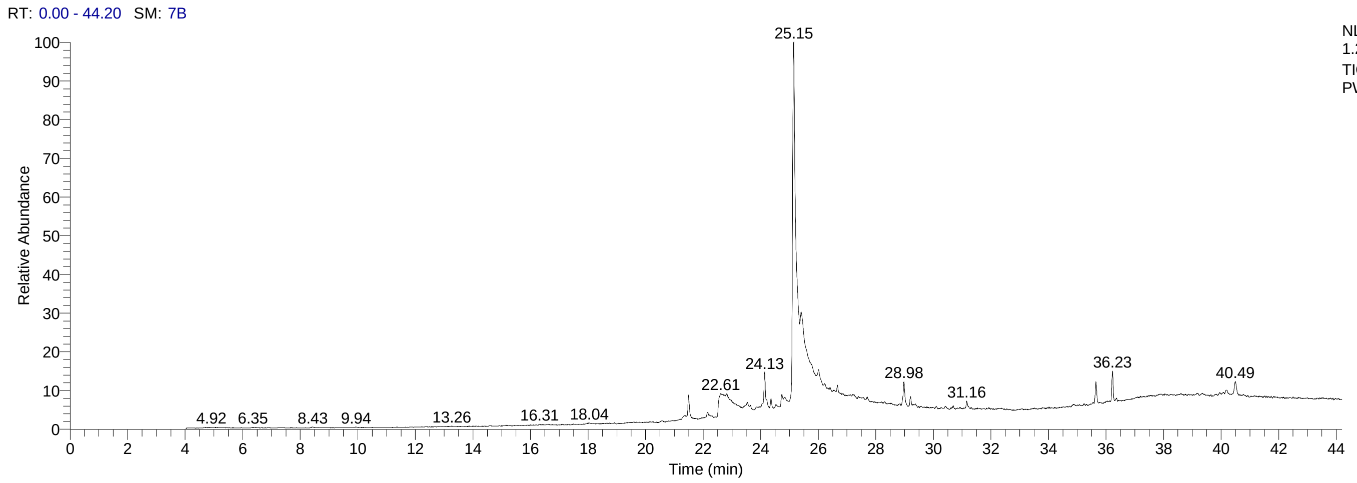

RT	Area %
21.50	3.02
22.61	8.90
22.82	4.40
24.13	4.82
24.36	0.94
24.73	1.60
25.14	56.59
25.42	3.52
26.01	1.75
26.66	0.91
28.98	3.60
29.21	1.08
35.65	3.08
36.23	3.45
40.49	2.35

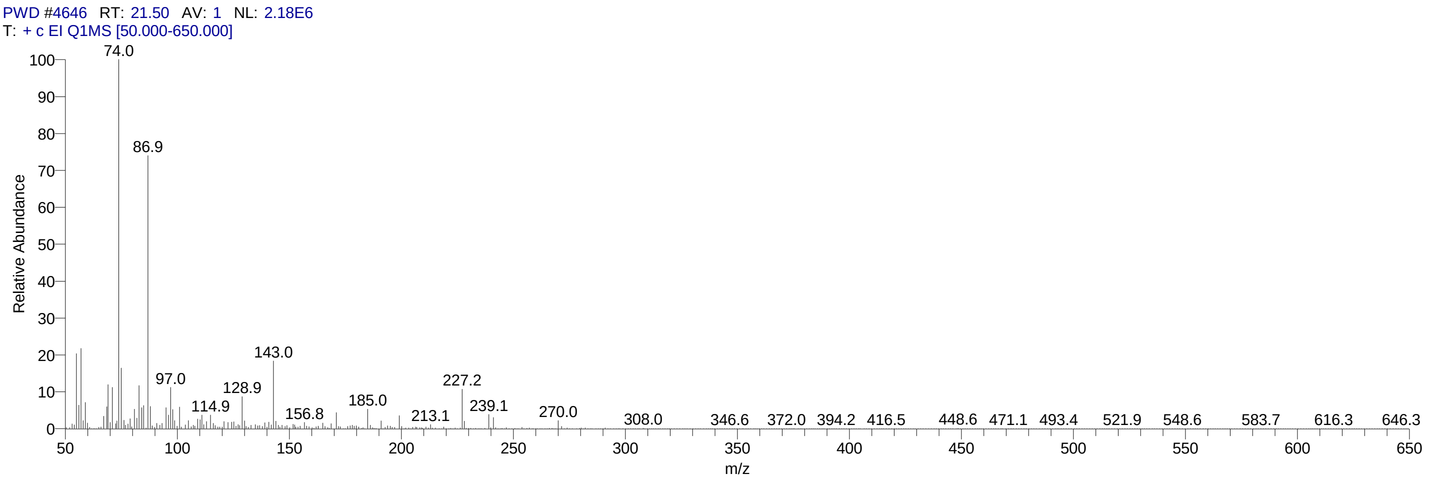

RT	Compound Name	Area %	Molecular Formula	Molecular Weight	Cas #	MF	Library
21.50	Hexadecanoic acid, methyl ester	3.02	C17H34O2	270	112-39-0	934	replib
21.50	PENTADECANOIC ACID, 14-METHYL-, METHYL ESTER	3.02	C17H34O2	270	5129-60-2	799	WileyRegistry8e
21.50	HEXADECANOIC ACID, METHYL ESTER	3.02	C17H34O2	270	112-39-0	803	WileyRegistry8e

My GC-MS Report

RT	Compound Name	Area %	Molecular Formula	Molecular Weight	Cas #	MF	Library
21.50	HEXADECANOIC ACID, METHYL ESTER	3.02	C17H34O2	270	112-39-0	835	WileyRegistry8e
21.50	Hexadecanoic acid, methyl ester	3.02	C17H34O2	270	112-39-0	839	replib

Compound Structure	Hit Spectrum

Hexadecanoic acid, methyl ester
Formula C17H34O2, MW 270, CAS# 112-39-0, Entry# 10415
Palmitic acid, methyl ester

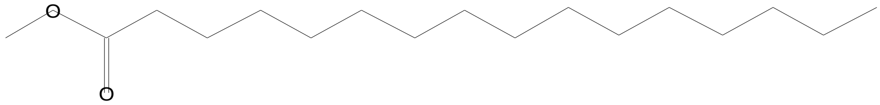

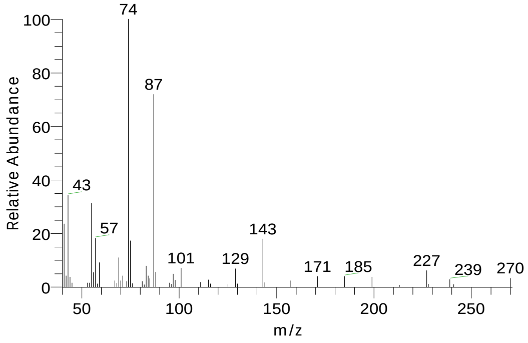

PENTADECANOIC ACID, 14-METHYL-, METHYL ESTER
Formula C17H34O2, MW 270, CAS# 5129-60-2, Entry# 161312
METHYL 14-METHYLPENTADECANOATE

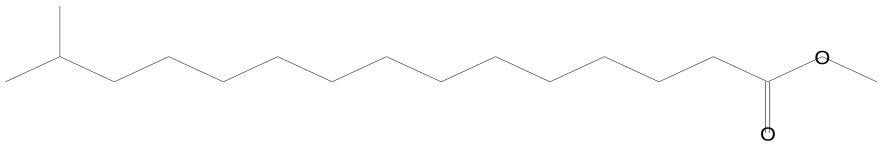

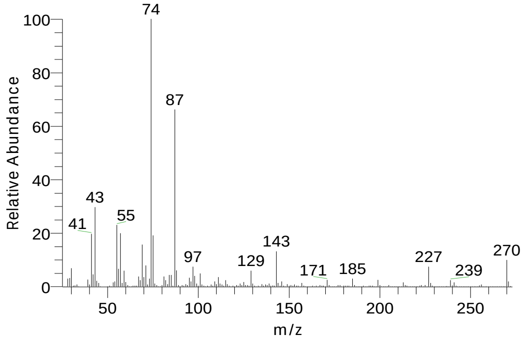

HEXADECANOIC ACID, METHYL ESTER
Formula C17H34O2, MW 270, CAS# 112-39-0, Entry# 161288
METHYL HEXADECANOATE

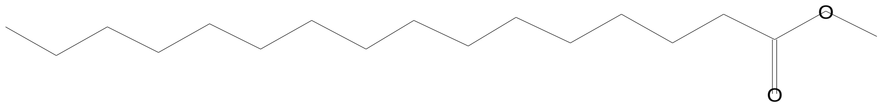

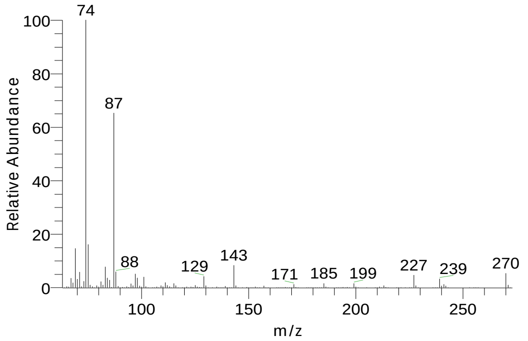

HEXADECANOIC ACID, METHYL ESTER
Formula C17H34O2, MW 270, CAS# 112-39-0, Entry# 161274
METHYL HEXADECANOATE

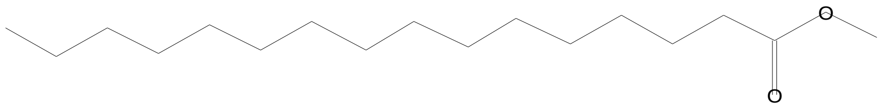

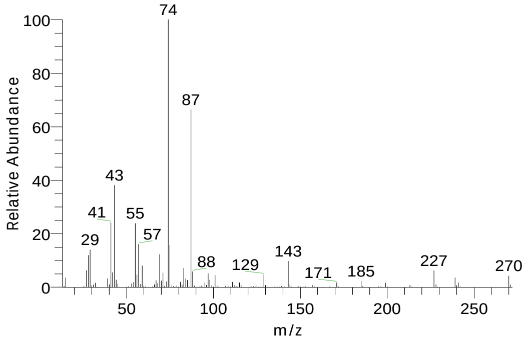

My GC-MS Report

Compound Structure | Hit Spectrum

Hexadecanoic acid, methyl ester
Formula C17H34O2, MW 270, CAS# 112-39-0, Entry# 10411
Palmitic acid, methyl ester

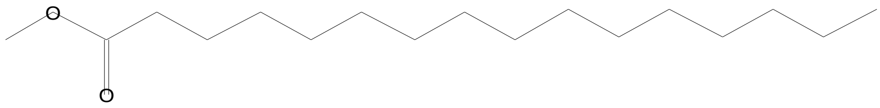

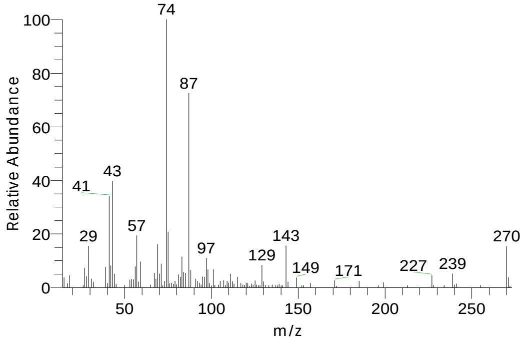

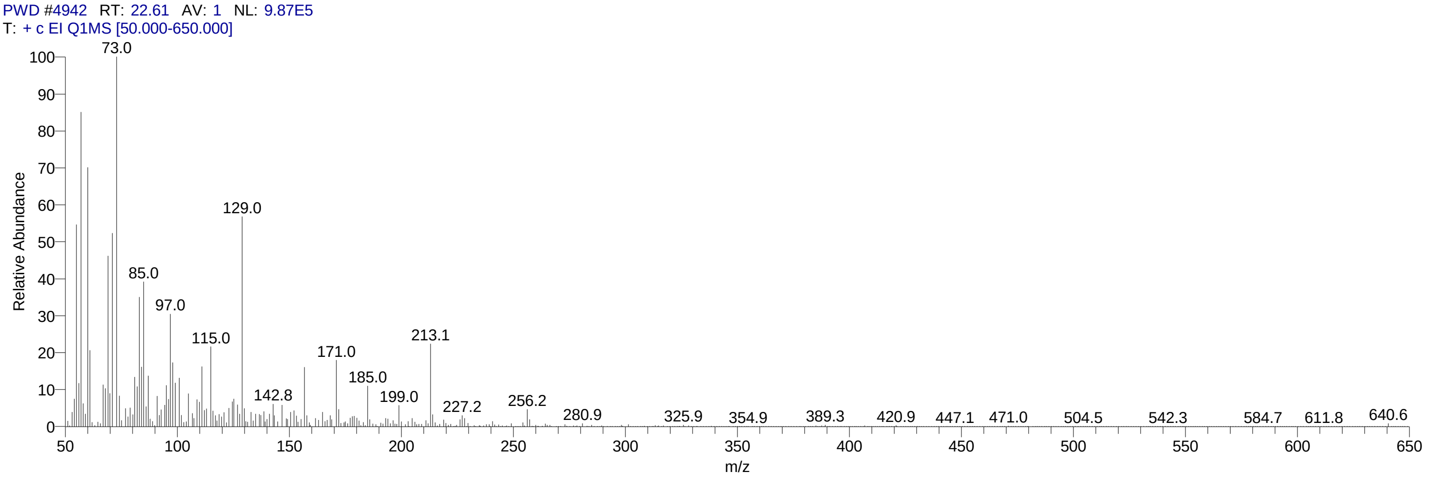

RT	Compound Name	Area %	Molecular Formula	Molecular Weight	Cas #	MF	Library
22.61	HEXADECANOIC ACID	8.90	C16H32O2	256	57-10-3	851	WileyRegistry8e
22.61	n-Hexadecanoic acid	8.90	C16H32O2	256	57-10-3	831	replib
22.61	HEXADECANOIC ACID	8.90	C16H32O2	256	57-10-3	821	WileyRegistry8e
22.61	n-Hexadecanoic acid	8.90	C16H32O2	256	57-10-3	820	mainlib
22.61	HEXADECANOIC ACID	8.90	C16H32O2	256	57-10-3	820	WileyRegistry8e

Compound Structure | Hit Spectrum

HEXADECANOIC ACID
Formula C16H32O2, MW 256, CAS# 57-10-3, Entry# 146746
HEXADECANOATE

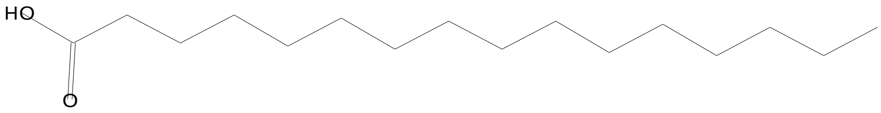

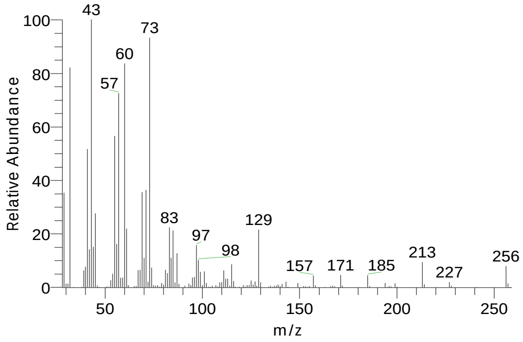

My GC-MS Report

Compound Structure | Hit Spectrum

n-Hexadecanoic acid
Formula C16H32O2, MW 256, CAS# 57-10-3, Entry# 7566
Hexadecanoic acid

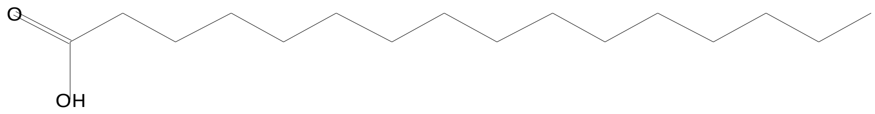

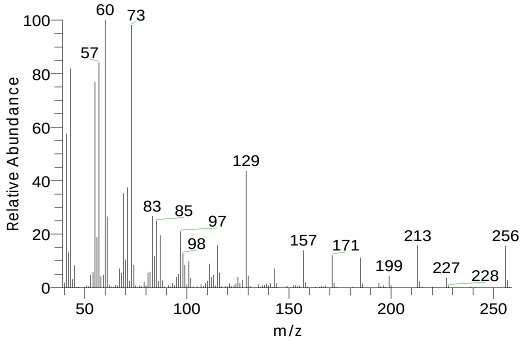

HEXADECANOIC ACID
Formula C16H32O2, MW 256, CAS# 57-10-3, Entry# 146743
HEXADECANOATE

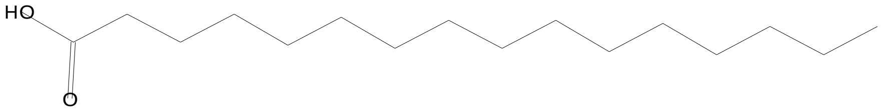

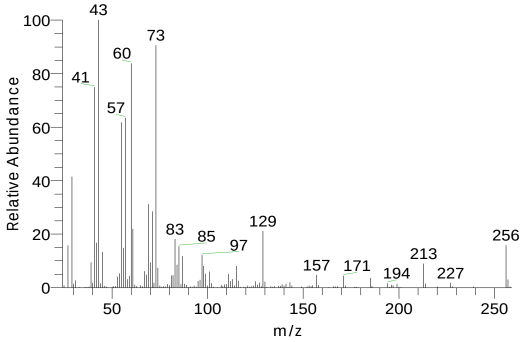

n-Hexadecanoic acid
Formula C16H32O2, MW 256, CAS# 57-10-3, Entry# 9208
Hexadecanoic acid

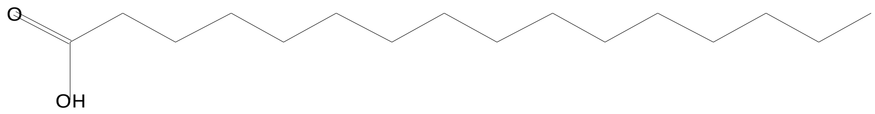

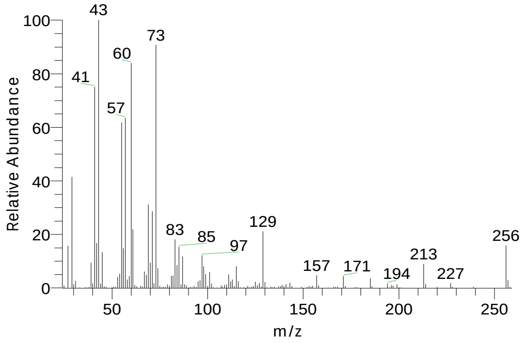

HEXADECANOIC ACID
Formula C16H32O2, MW 256, CAS# 57-10-3, Entry# 387074
HEXADECANOATE

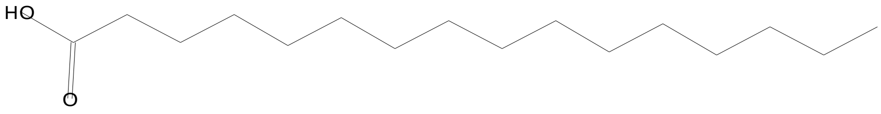

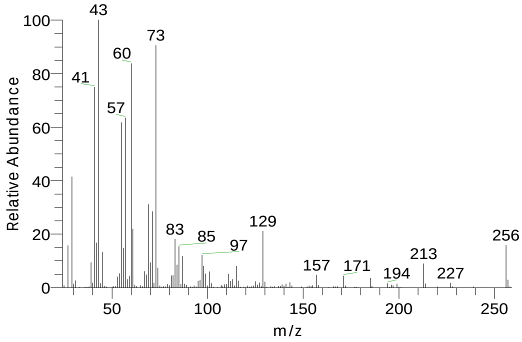

My GC-MS Report

PWD #4998 RT: 22.82 AV: 1 NL: 7.40E5
T: + c EI Q1MS [50.000-650.000]

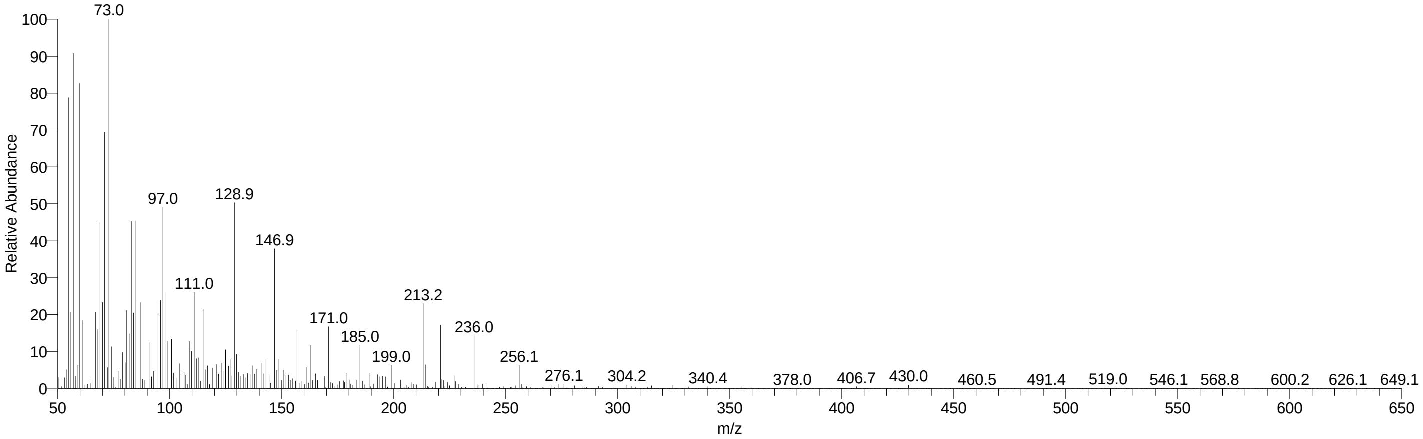

RT	Compound Name	Area %	Molecular Formula	Molecular Weight	Cas #	MF	Library
22.82	HEXADECANOIC ACID, 2,3-DIHYDROXYPROPYL ESTER	4.40	C19H38O4	330	542-44-9	732	WileyRegistry8e
22.82	Estra-1,3,5(10)-trien-17á-ol	4.40	C18H24O	256	2529-64-8	776	mainlib
22.82	PENTADECANOIC ACID	4.40	C15H30O2	242	1002-84-2	750	WileyRegistry8e
22.82	HEXADECANOIC ACID	4.40	C16H32O2	256	57-10-3	826	WileyRegistry8e
22.82	9-OCTADECENOIC ACID (Z)-	4.40	C18H34O2	282	112-80-1	894	WileyRegistry8e

Compound Structure | Hit Spectrum

HEXADECANOIC ACID, 2,3-DIHYDROXYPROPYL ESTER
Formula C19H38O4, MW 330, CAS# 542-44-9, Entry# 214589
2,3-DIHYDROXYPROPYL PALMITATE #

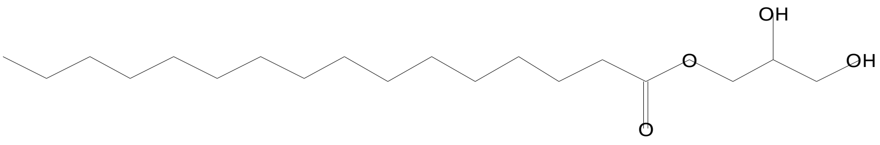

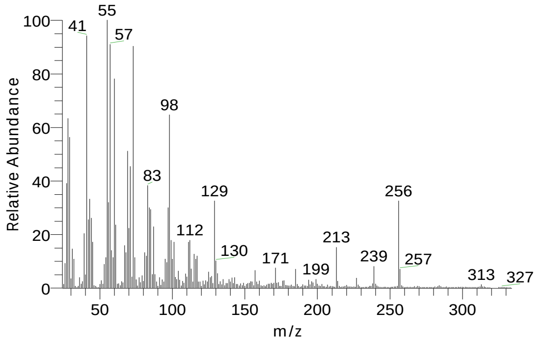

Estra-1,3,5(10)-trien-17á-ol
Formula C18H24O, MW 256, CAS# 2529-64-8, Entry# 7736
Estra-1,3,5(10)-trien-17-ol, (17á)-

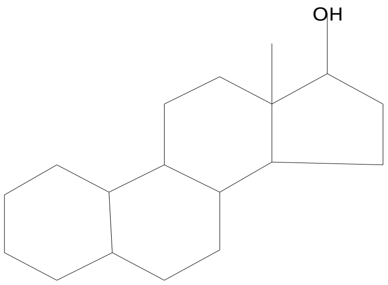

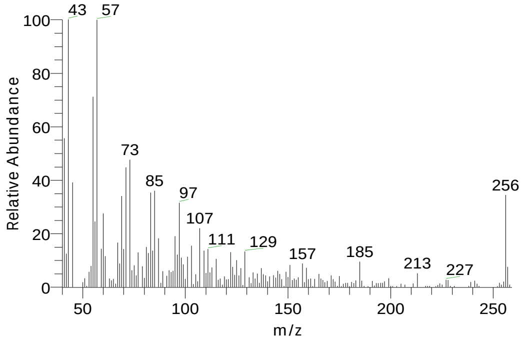

My GC-MS Report

Compound Structure | Hit Spectrum

PENTADECANOIC ACID
Formula C15H30O2, MW 242, CAS# 1002-84-2, Entry# 131990
14FA

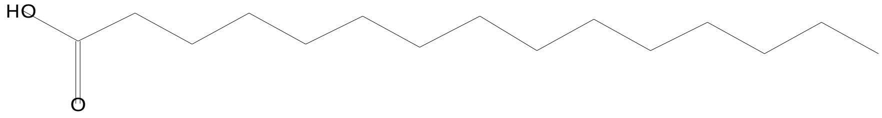

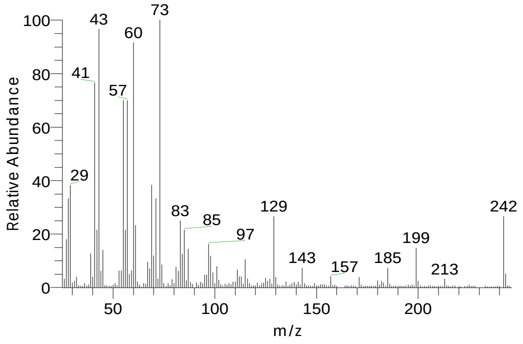

HEXADECANOIC ACID
Formula C16H32O2, MW 256, CAS# 57-10-3, Entry# 146746
HEXADECANOATE

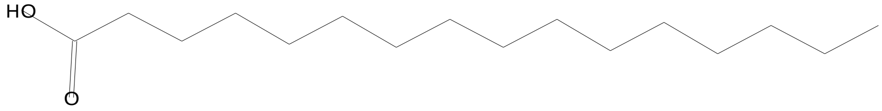

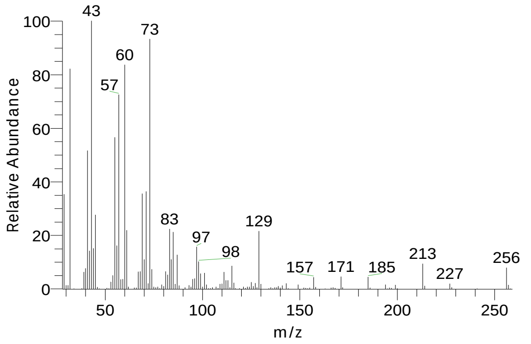

9-OCTADECENOIC ACID (Z)-
Formula C18H34O2, MW 282, CAS# 112-80-1, Entry# 172901
OCTADEC-9-ENOIC ACID

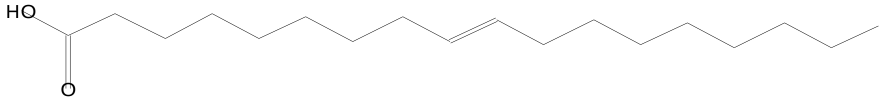

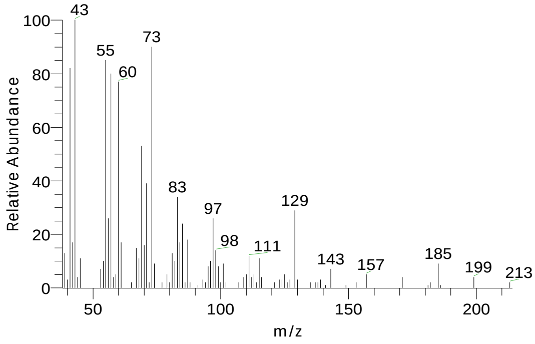

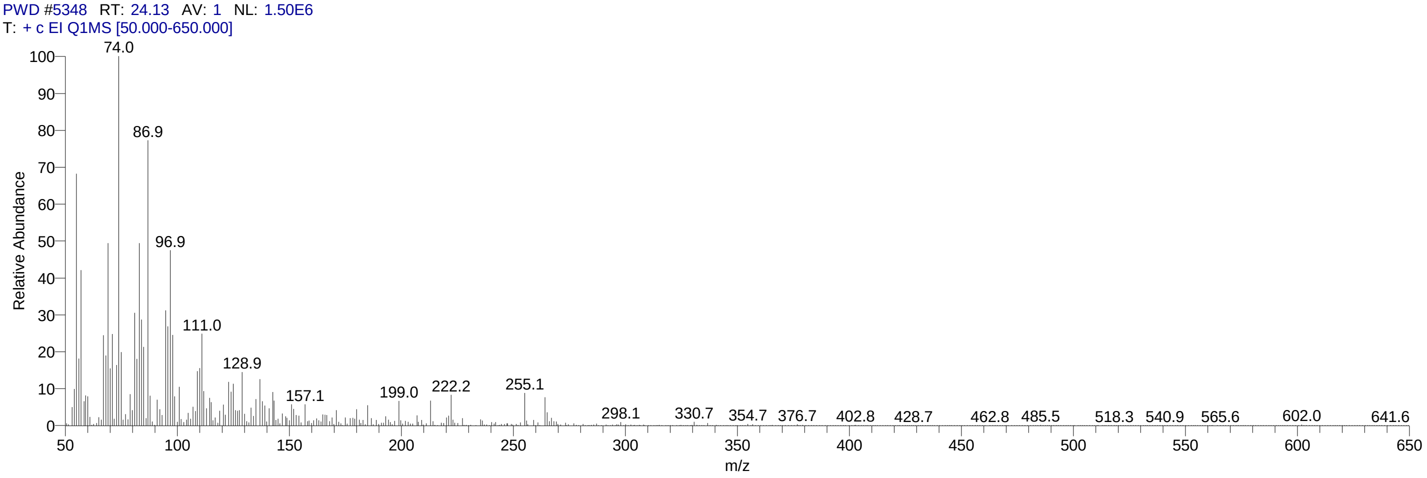

RT	Compound Name	Area %	Molecular Formula	Molecular Weight	Cas #	MF	Library
24.13	17-OCTADECENOIC ACID, METHYL ESTER	4.82	C19H36O2	296	18654-84-7	810	WileyRegistry8e
24.13	ELAIDINSAEUREMETHYLESTER	4.82	C19H36O2	296	NA	826	WileyRegistry8e
24.13	10-Octadecenoic acid, methyl ester	4.82	C19H36O2	296	13481-95-3	804	mainlib

My GC-MS Report

RT	Compound Name	Area %	Molecular Formula	Molecular Weight	Cas #	MF	Library
24.13	10-OCTADECENOIC ACID, METHYL ESTER	4.82	C19H36O2	296	13481-95-3	804	WileyRegistry8e
24.13	16-OCTADECENOIC ACID, METHYL ESTER	4.82	C19H36O2	296	56554-49-5	803	WileyRegistry8e

Compound Structure | Hit Spectrum

17-OCTADECENOIC ACID, METHYL ESTER
Formula C19H36O2, MW 296, CAS# 18654-84-7, Entry# 186184
METHYL CIS-OCTADECA-17-ENOATE

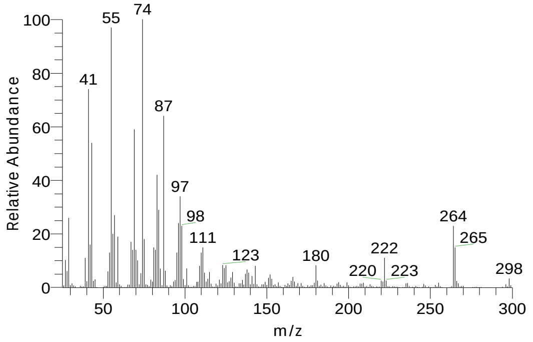

ELAIDINSAEUREMETHYLESTER
Formula C19H36O2, MW 296, CAS# NA, Entry# 359292
METHYL 9-OCTADECENOATE

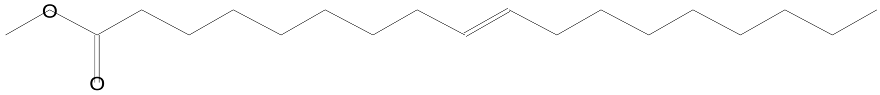

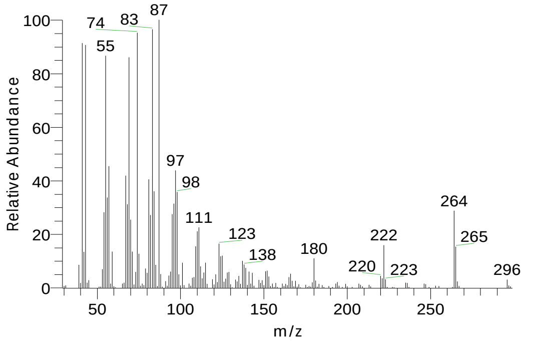

10-Octadecenoic acid, methyl ester
Formula C19H36O2, MW 296, CAS# 13481-95-3, Entry# 19319
Methyl 10-octadecenoate

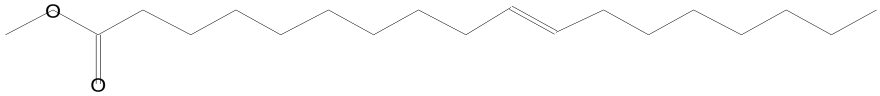

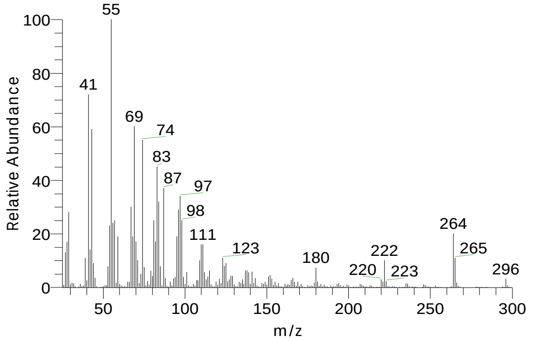

10-OCTADECENOIC ACID, METHYL ESTER
Formula C19H36O2, MW 296, CAS# 13481-95-3, Entry# 186173
METHYL OCTADEC-10-ENOATE

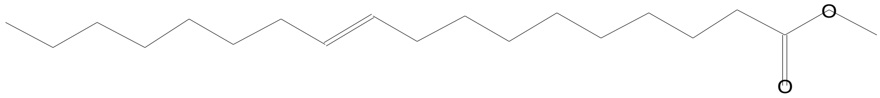

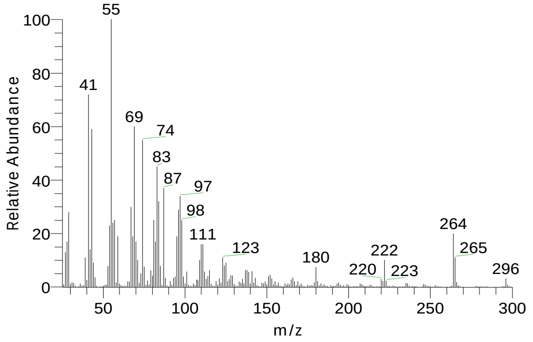

My GC-MS Report

Compound Structure | Hit Spectrum

16-OCTADECENOIC ACID, METHYL ESTER
Formula C19H36O2, MW 296, CAS# 56554-49-5, Entry# 186182
METHYL OCTADEC-16-ENOATE

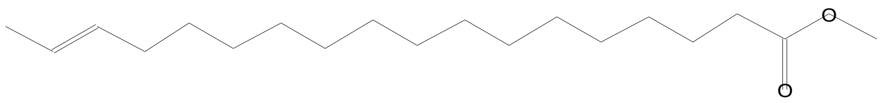

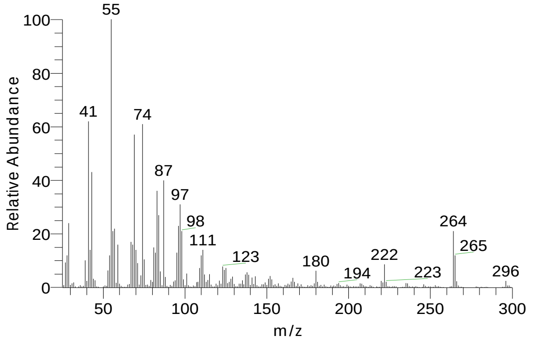

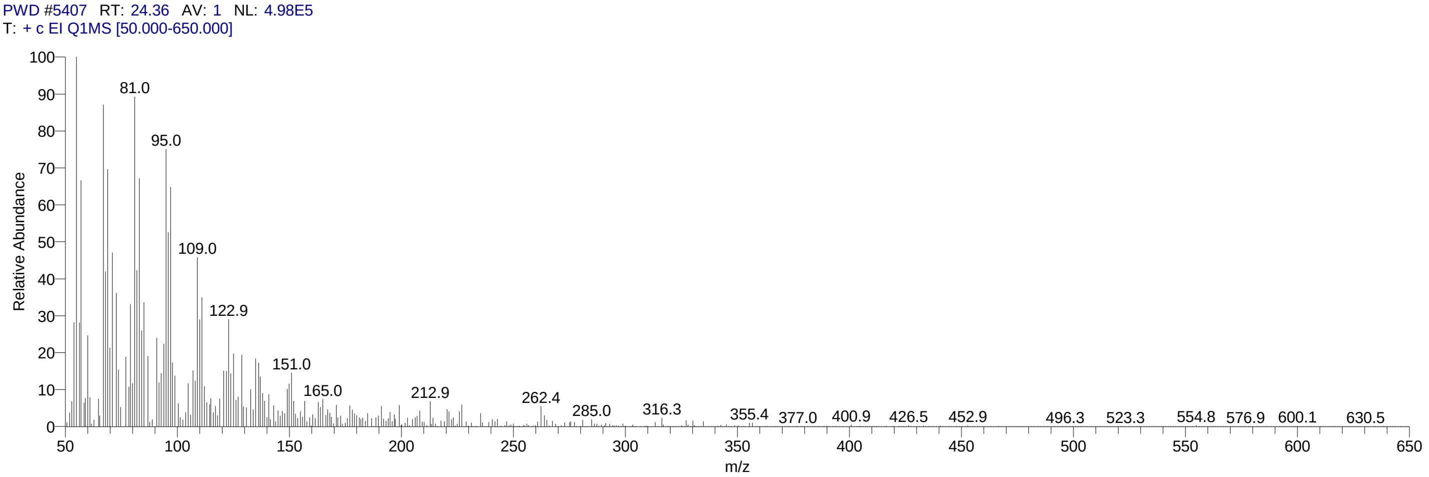

RT	Compound Name	Area %	Molecular Formula	Molecular Weight	Cas #	MF	Library
24.36	E,E,Z-1,3,12-Nonadecatriene-5,14-diol	0.94	C19H34O2	294	NA	777	mainlib
24.36	CYCLOPROPANEOCTANOIC ACID, 2-[[2-[(2-ETHYLCYCLOPROPYL)METHYL]CYCLOPROPYL]METHYL]-, METHYL ESTER	0.94	C22H38O2	334	10152-71-3	808	WileyRegistry8e
24.36	17-Octadecynoic acid	0.94	C18H32O2	280	34450-18-5	777	mainlib
24.36	Cyclopropaneoctanoic acid, 2-[[2-[(2-ethylcyclopropyl)methyl]cyclopropyl]methyl]-, methyl ester	0.94	C22H38O2	334	10152-71-3	809	mainlib
24.36	9,12-Octadecadienoic acid (Z,Z)-	0.94	C18H32O2	280	60-33-3	788	replib

Compound Structure | Hit Spectrum

E,E,Z-1,3,12-Nonadecatriene-5,14-diol
Formula C19H34O2, MW 294, CAS# NA, Entry# 21026
(3E,12Z)-1,3,12-Nonadecatriene-5,14-diol #

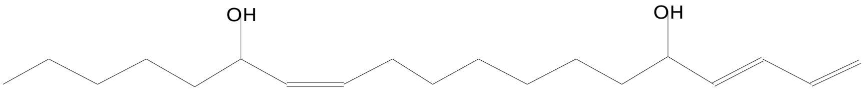

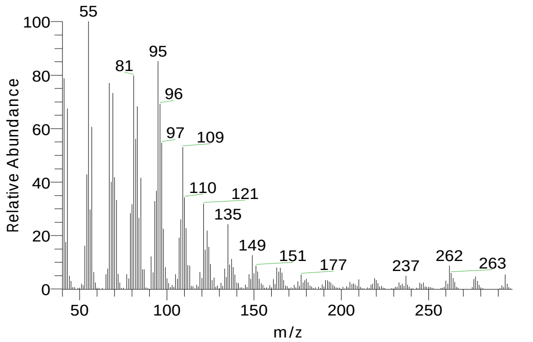

My GC-MS Report

Compound Structure | Hit Spectrum

Formula C22H38O2, MW 334, CAS# 10152-71-3, Entry# 217974

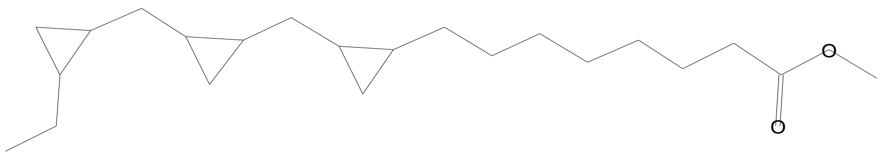

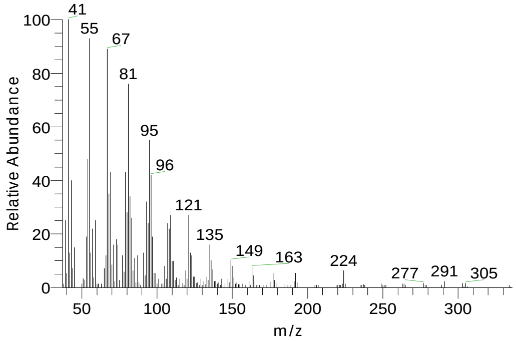

17-Octadecynoic acid
Formula C18H32O2, MW 280, CAS# 34450-18-5, Entry# 20510
$:28DZIILFGADWDKMF-UHFFFAOYSA-N

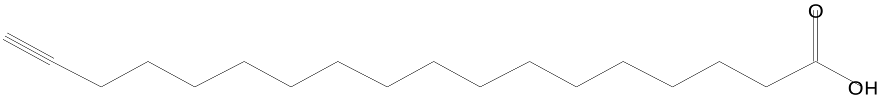

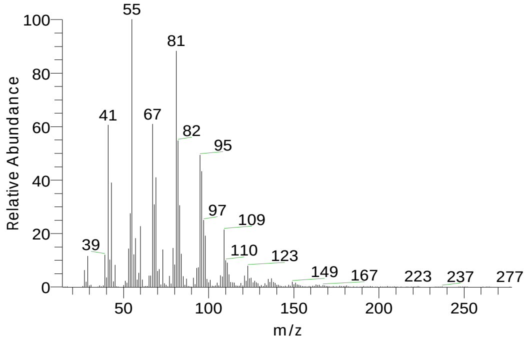

Formula C22H38O2, MW 334, CAS# 10152-71-3, Entry# 2765
Methyl 8-[2-((2-[(2-ethylcyclopropyl)methyl]cyclopropyl)methyl)cyclopropyl]octanoate #

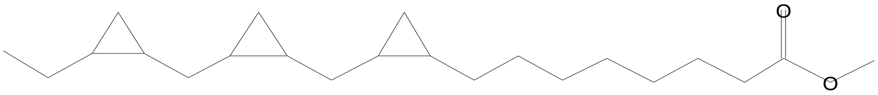

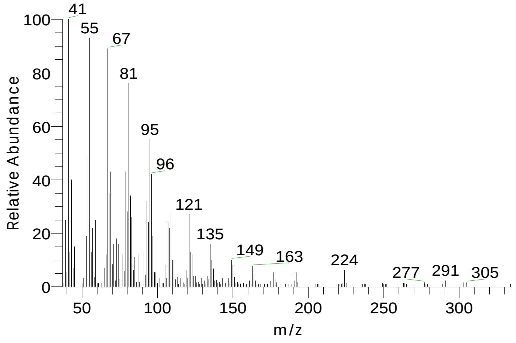

9,12-Octadecadienoic acid (Z,Z)-
Formula C18H32O2, MW 280, CAS# 60-33-3, Entry# 8057
cis-9,cis-12-Octadecadienoic acid

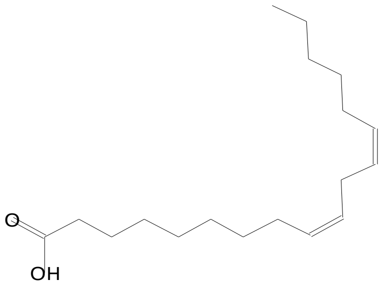

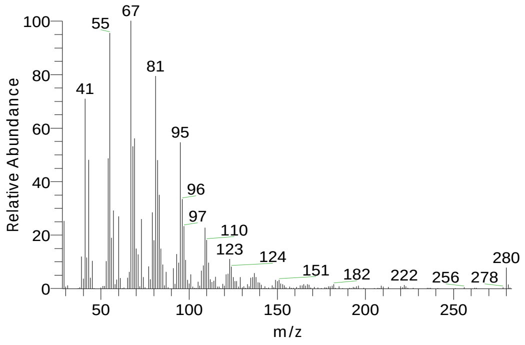

My GC-MS Report

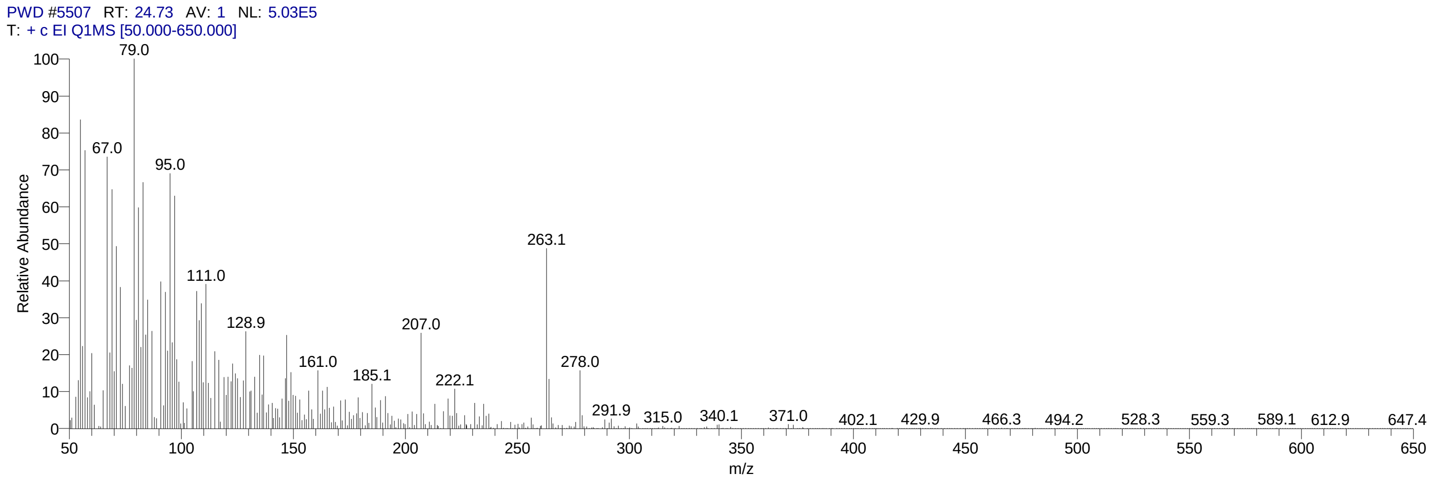

RT	Compound Name	Area %	Molecular Formula	Molecular Weight	Cas #	MF	Library
24.73	1H,4H,5H,8H-3A,4A,7A,8A-TETRAAZACYCLOPENTA[DEF]FLUORENE, HEXAHYDRO-2,2,4,6,6,8-HEXAMETHYL-, (4à,8à,8Bà,8C.A LPHA.)-	1.60	C16H30N4	278	122763-31-9	889	WileyRegistry8e
24.73	4-PENTEN-1-ONE, 2-[BIS(METHYLTHIO)METHYLENE]-4-METHYL-1-PHENYL-	1.60	C15H18OS2	278	116145-44-9	845	WileyRegistry8e
24.73	(+-)-5-HYDROXY-6-(1-HYDROXYETHYL)-2,7-DIMETHOXYNAPHTHOQUINONE	1.60	C14H14O6	278	NA	841	WileyRegistry8e
24.73	9,12-Octadecadienoic acid (Z,Z)-, 2-hydroxy-1-(hydroxymethyl)ethyl ester	1.60	C21H38O4	354	3443-82-1	720	replib
24.73	Butyl 9,12,15-octadecatrienoate	1.60	C22H38O2	334	NA	743	mainlib

Compound Structure

Hit Spectrum

Formula C16H30N4, MW 278, CAS# 122763-31-9, Entry# 168749

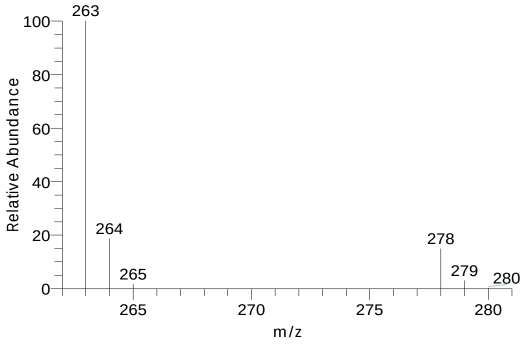

4-PENTEN-1-ONE, 2-[BIS(METHYLTHIO)METHYLENE]-4-METHYL-1-PHENYL-
Formula C15H18OS2, MW 278, CAS# 116145-44-9, Entry# 168414
2-BIS(METHYLTHIO)METHYLENE-1-PHRENYL-4-METHYL-4-PENTEN-1-ONE

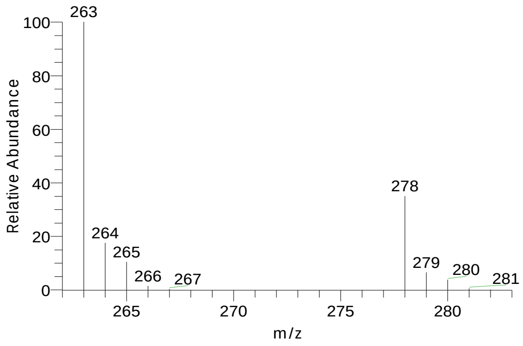

My GC-MS Report

Compound Structure | Hit Spectrum

(+-)-5-HYDROXY-6-(1-HYDROXYETHYL)-2,7-DIMETHOXYNAPHTHOQUINONE
Formula C14H14O6, MW 278, CAS# NA, Entry# 168333

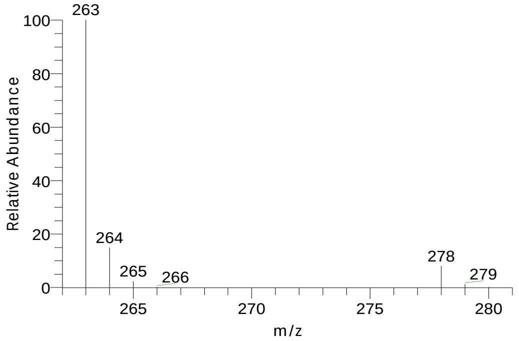

9,12-Octadecadienoic acid (Z,Z)-, 2-hydroxy-1-(hydroxymethyl)ethyl ester
Formula C21H38O4, MW 354, CAS# 3443-82-1, Entry# 8055
Linolein, 2-mono-

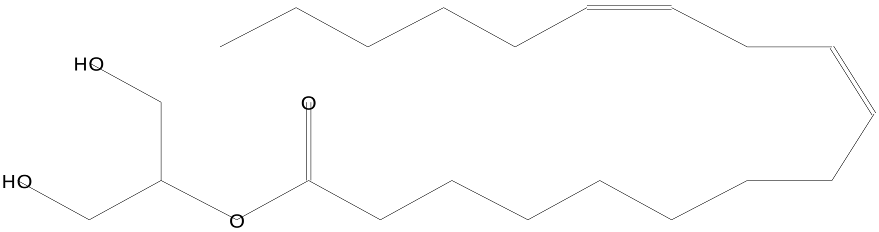

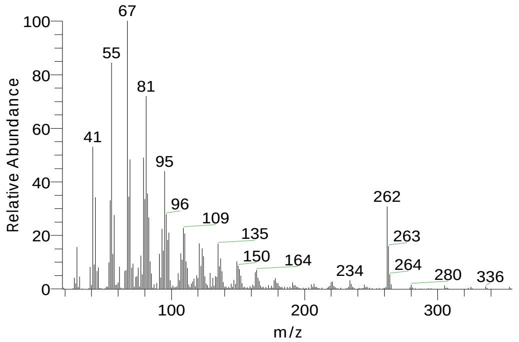

Butyl 9,12,15-octadecatrienoate
Formula C22H38O2, MW 334, CAS# NA, Entry# 48702
$:28QQNROWCMALTXCD-XQOKXTRKSA-N

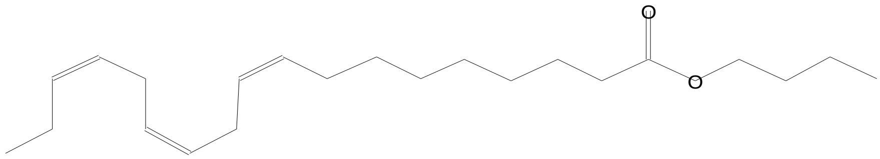

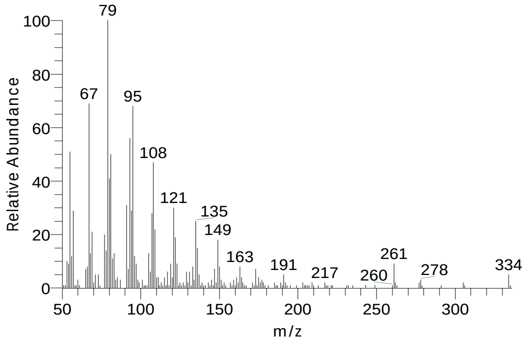

PWD #5616 RT: 25.14 AV: 1 NL: 9.61E6
T: + c EI Q1MS [50.000-650.000]

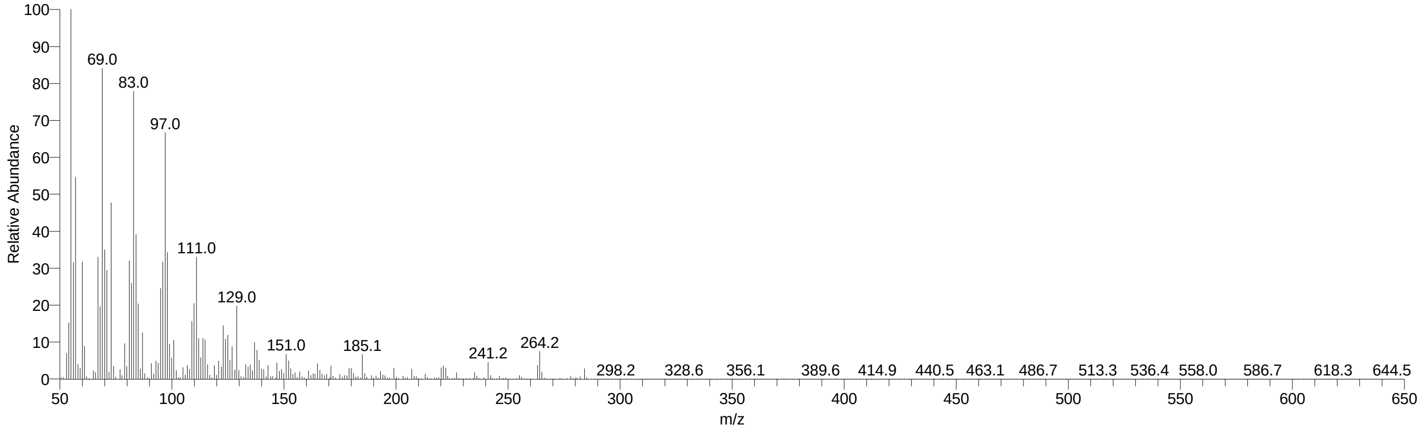

RT	Compound Name	Area %	Molecular Formula	Molecular Weight	Cas #	MF	Library
25.14	9-Octadecenoic acid, (E)-	56.59	C18H34O2	282	112-79-8	901	replib
25.14	Oleic Acid	56.59	C18H34O2	282	112-80-1	898	replib
25.14	9-OCTADECENOIC ACID (Z)-	56.59	C18H34O2	282	112-80-1	892	WileyRegistry8e
25.14	cis-13-Octadecenoic acid	56.59	C18H34O2	282	13126-39-1	882	mainlib

My GC-MS Report

RT	Compound Name	Area %	Molecular Formula	Molecular Weight	Cas #	MF	Library
25.14	trans-13-Octadecenoic acid	56.59	C18H34O2	282	693-71-0	880	mainlib

Compound Structure | Hit Spectrum

9-Octadecenoic acid, (E)-
Formula C18H34O2, MW 282, CAS# 112-79-8, Entry# 5015
trans-ë(sup 9)-Octadecenoic acid

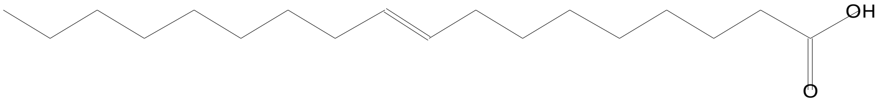

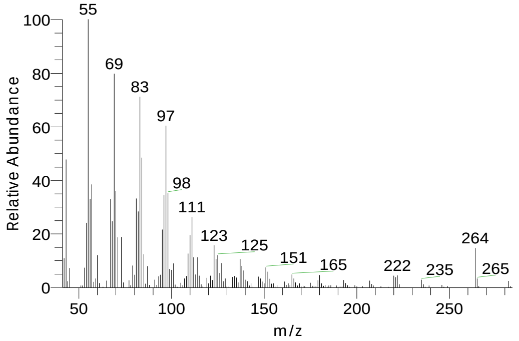

SI 875, RSI 898, replib, Entry# 5017, CAS# 112-80-1, Oleic Acid

Oleic Acid
Formula C18H34O2, MW 282, CAS# 112-80-1, Entry# 5017
9-Octadecenoic acid (Z)-

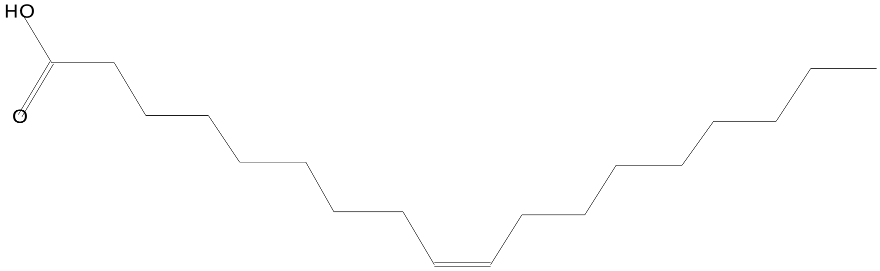

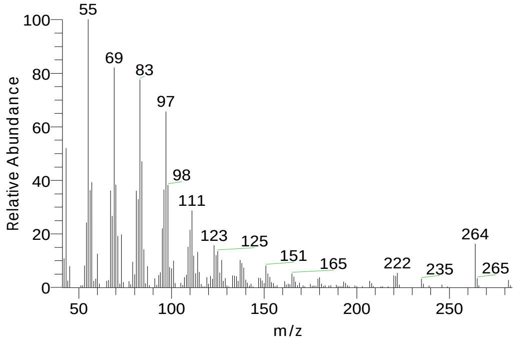

9-OCTADECENOIC ACID (Z)-
Formula C18H34O2, MW 282, CAS# 112-80-1, Entry# 172902
OCTADEC-9-ENOIC ACID

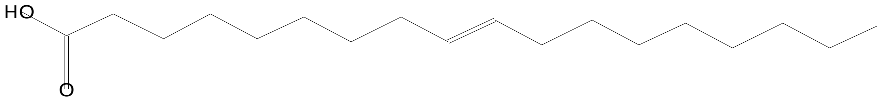

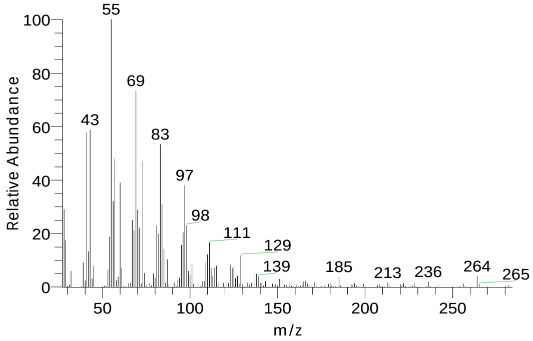

cis-13-Octadecenoic acid
Formula C18H34O2, MW 282, CAS# 13126-39-1, Entry# 20126
$:28BDLLSHRIFPDGQB-WAYWQWQTSA-N

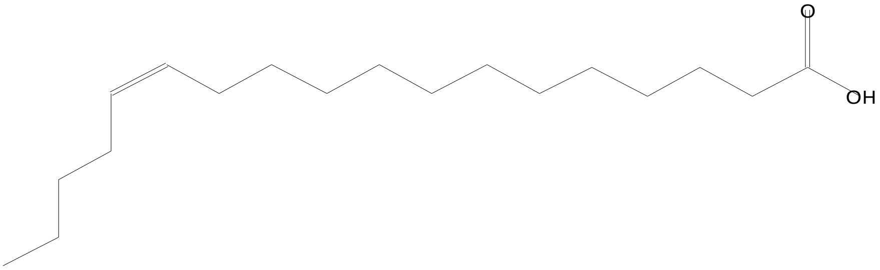

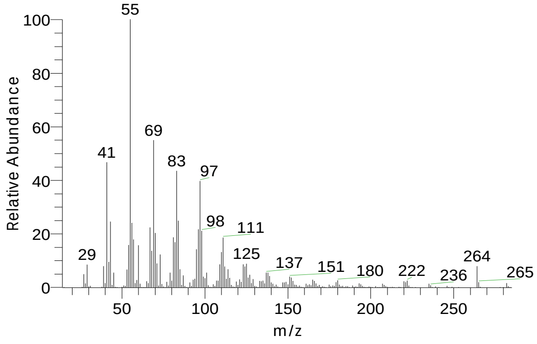

My GC-MS Report

Compound Structure | Hit Spectrum

trans-13-Octadecenoic acid
Formula C18H34O2, MW 282, CAS# 693-71-0, Entry# 19306
$:28BDLLSHRIFPDGQB-AATRIKPKSA-N

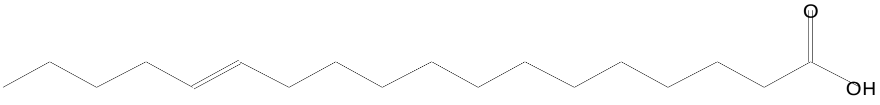

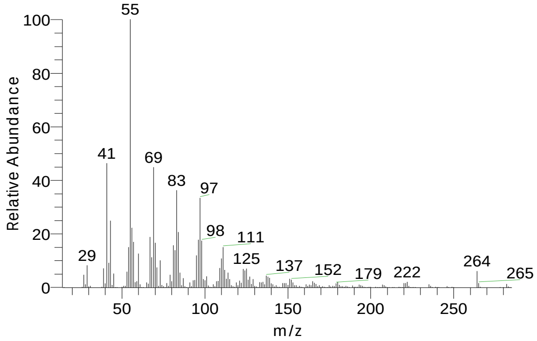

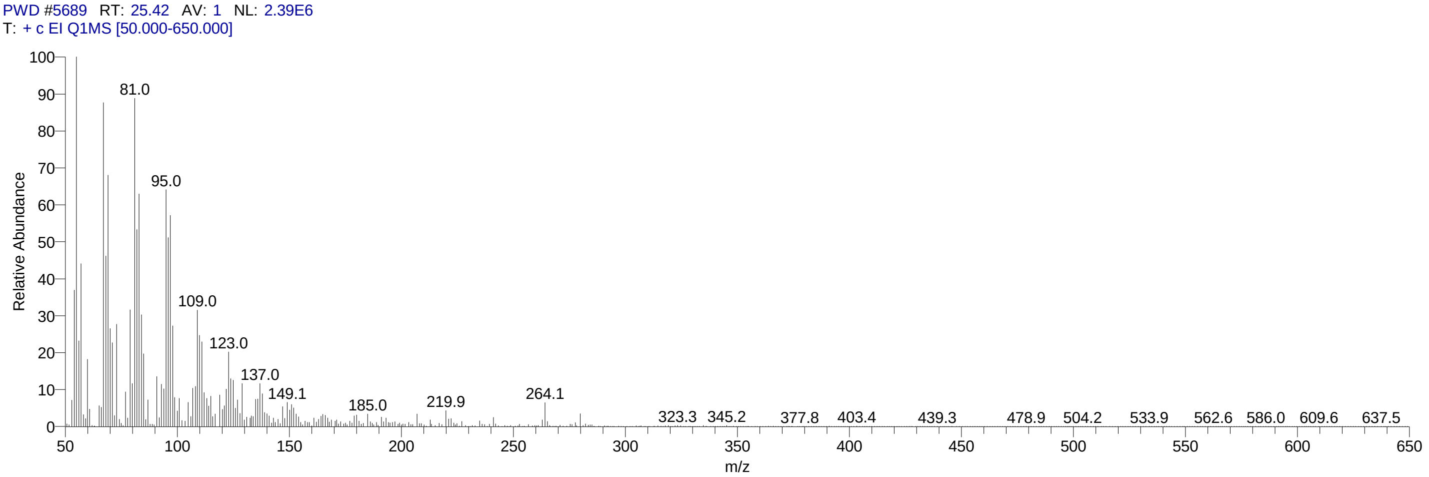

RT	Compound Name	Area %	Molecular Formula	Molecular Weight	Cas #	MF	Library
25.42	9,12-Octadecadienoic acid (Z,Z)-	3.52	C18H32O2	280	60-33-3	855	replib
25.42	9,12-OCTADECADIENOIC ACID (Z,Z)-	3.52	C18H32O2	280	60-33-3	854	WileyRegistry8e
25.42	17-Octadecynoic acid	3.52	C18H32O2	280	34450-18-5	828	mainlib
25.42	9,12-Octadecadienoyl chloride, (Z,Z)-	3.52	C18H31ClO	298	7459-33-8	842	replib
25.42	(9E,12E)-9,12-OCTADECADIENOYL CHLORIDE #	3.52	C18H31ClO	298	7459-33-8	842	WileyRegistry8e

Compound Structure | Hit Spectrum

9,12-Octadecadienoic acid (Z,Z)-
Formula C18H32O2, MW 280, CAS# 60-33-3, Entry# 8057
cis-9,cis-12-Octadecadienoic acid

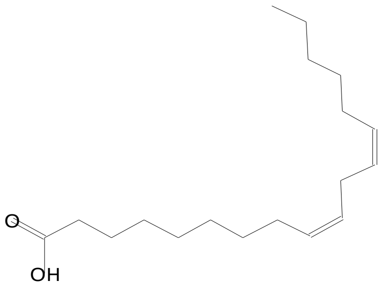

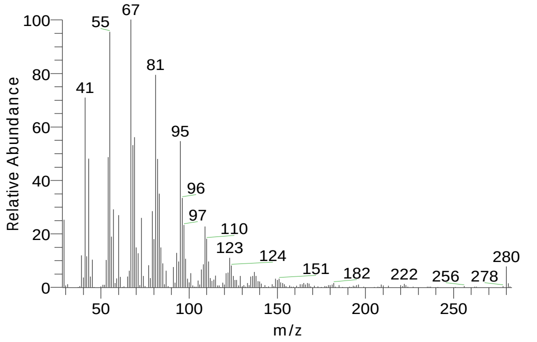

My GC-MS Report

Compound Structure | Hit Spectrum

9,12-OCTADECADIENOIC ACID (Z,Z)-
Formula C18H32O2, MW 280, CAS# 60-33-3, Entry# 170904
(9E,12E)-9,12-OCTADECADIENOIC ACID #

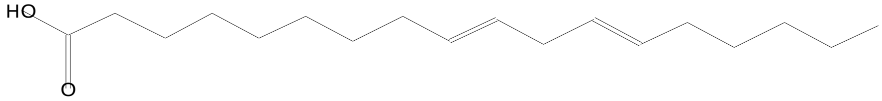

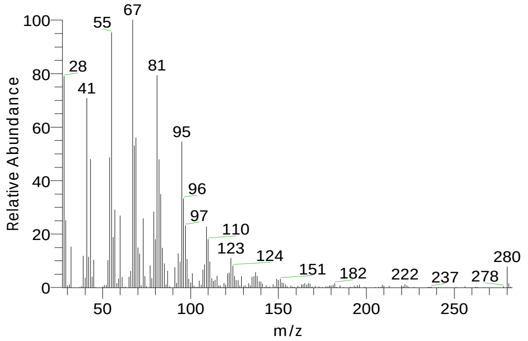

17-Octadecynoic acid
Formula C18H32O2, MW 280, CAS# 34450-18-5, Entry# 20510
$:28DZIILFGADWDKMF-UHFFFAOYSA-N

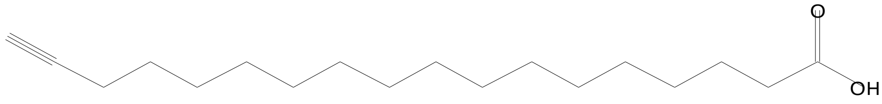

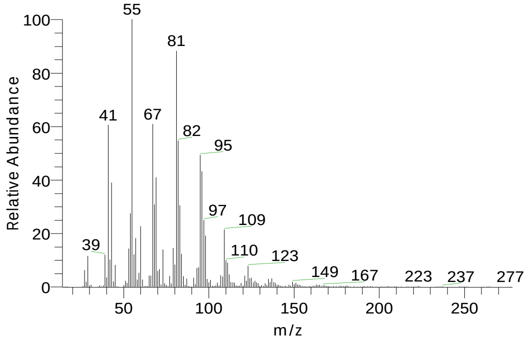

9,12-Octadecadienoyl chloride, (Z,Z)-
Formula C18H31ClO, MW 298, CAS# 7459-33-8, Entry# 4940
Linoleoyl chloride

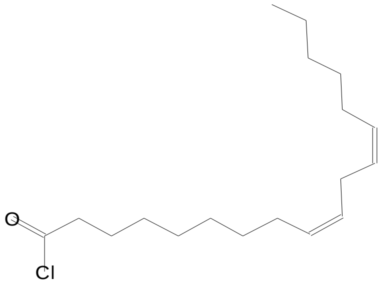

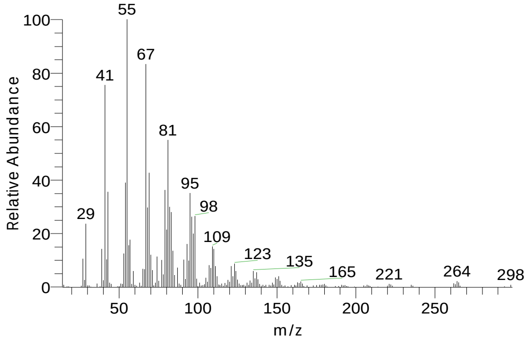

(9E,12E)-9,12-OCTADECADIENOYL CHLORIDE #
Formula C18H31ClO, MW 298, CAS# 7459-33-8, Entry# 187801
(9E,12E)-9,12-OCTADECADIENOYL CHLORIDE

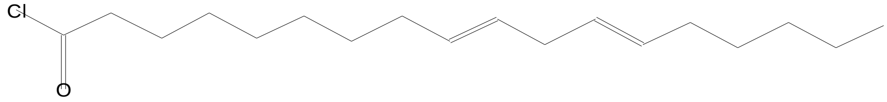

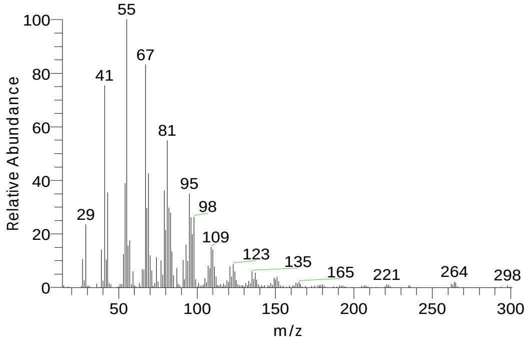

My GC-MS Report

PWD #5848 RT: 26.01 AV: 1 NL: 1.13E6
T: + c EI Q1MS [50.000-650.000]

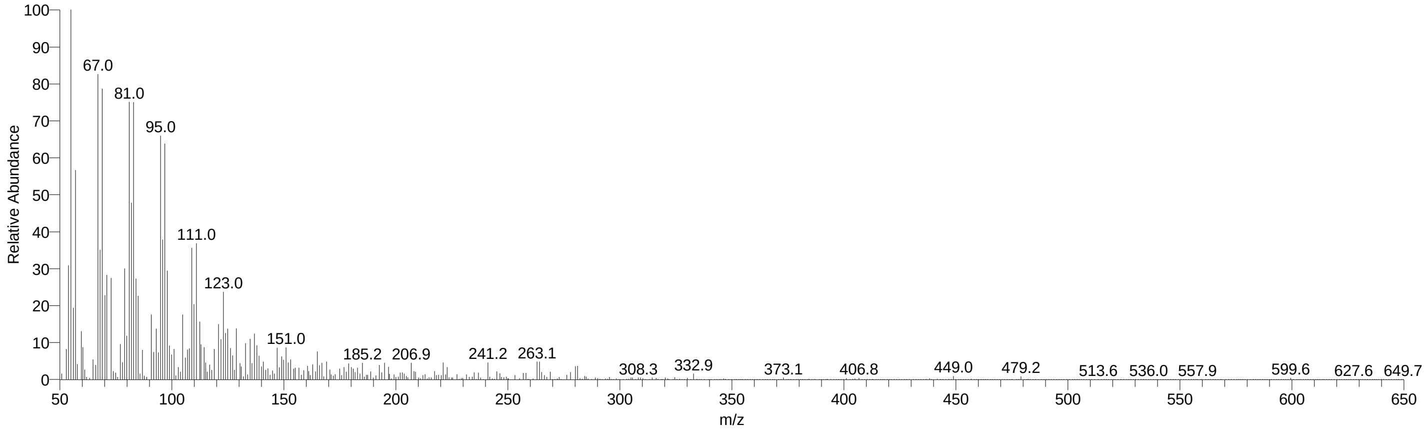

RT	Compound Name	Area %	Molecular Formula	Molecular Weight	Cas #	MF	Library
26.01	9-OCTADECENOIC ACID (Z)-	1.75	C18H34O2	282	112-80-1	799	WileyRegistry8e
26.01	trans-13-Octadecenoic acid	1.75	C18H34O2	282	693-71-0	804	mainlib
26.01	cis-Vaccenic acid	1.75	C18H34O2	282	506-17-2	796	mainlib
26.01	E,E,Z-1,3,12-Nonadecatriene-5,14-diol	1.75	C19H34O2	294	NA	783	mainlib
26.01	cis-13-Octadecenoic acid	1.75	C18H34O2	282	13126-39-1	796	mainlib

Compound Structure | Hit Spectrum

9-OCTADECENOIC ACID (Z)-
Formula C18H34O2, MW 282, CAS# 112-80-1, Entry# 172910
OCTADEC-9-ENOIC ACID

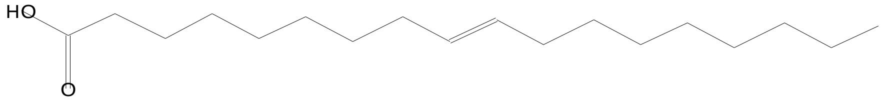

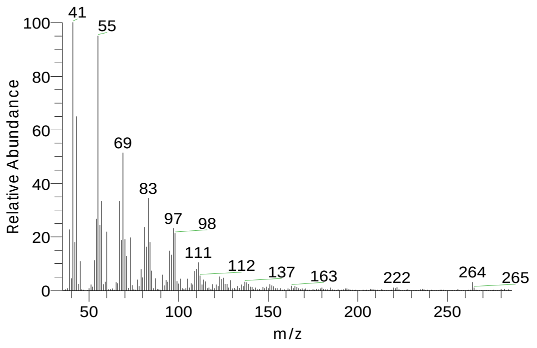

trans-13-Octadecenoic acid
Formula C18H34O2, MW 282, CAS# 693-71-0, Entry# 19306
$:28BDLLSHRIFPDGQB-AATRIKPKSA-N

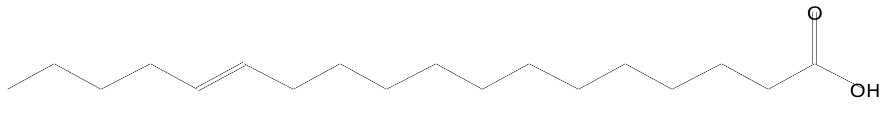

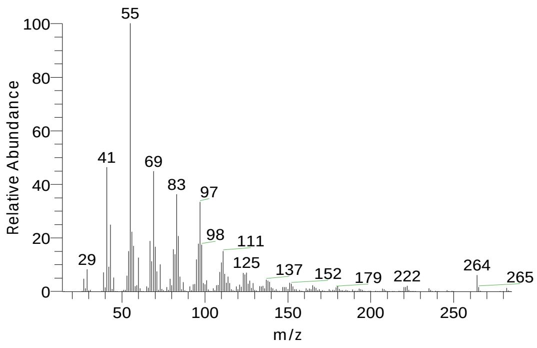

My GC-MS Report

Compound Structure | Hit Spectrum

cis-Vaccenic acid
Formula C18H34O2, MW 282, CAS# 506-17-2, Entry# 20090
11-Octadecenoic acid, (Z)-

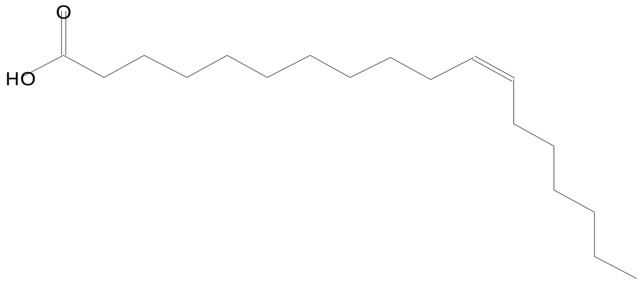

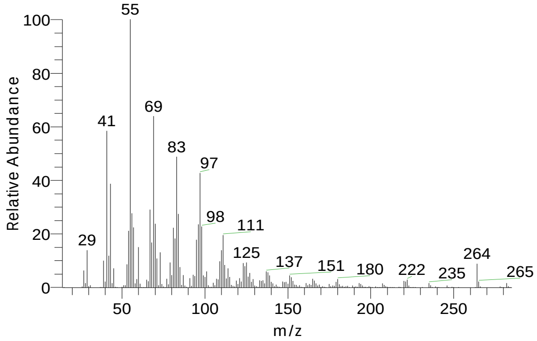

E,E,Z-1,3,12-Nonadecatriene-5,14-diol
Formula C19H34O2, MW 294, CAS# NA, Entry# 21026
(3E,12Z)-1,3,12-Nonadecatriene-5,14-diol #

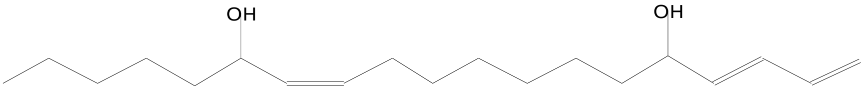

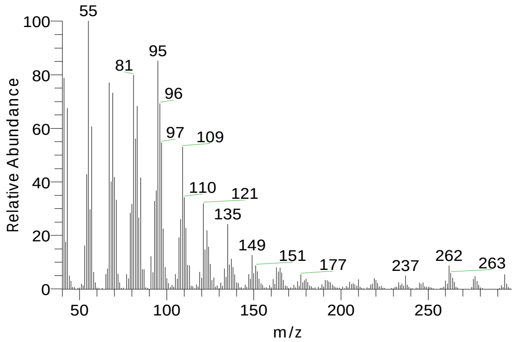

cis-13-Octadecenoic acid
Formula C18H34O2, MW 282, CAS# 13126-39-1, Entry# 20126
$:28BDLLSHRIFPDGQB-WAYWQWQTSA-N

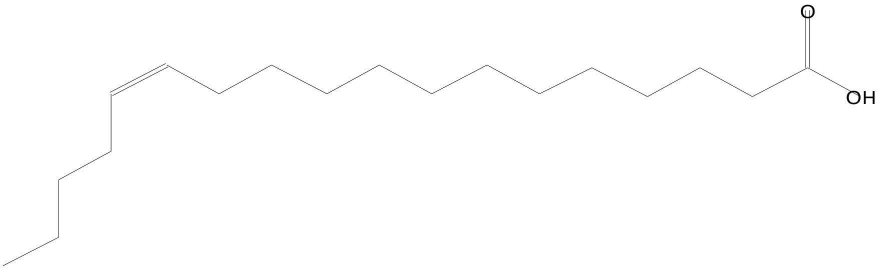

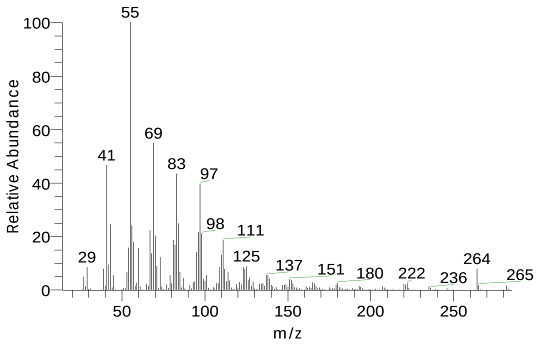

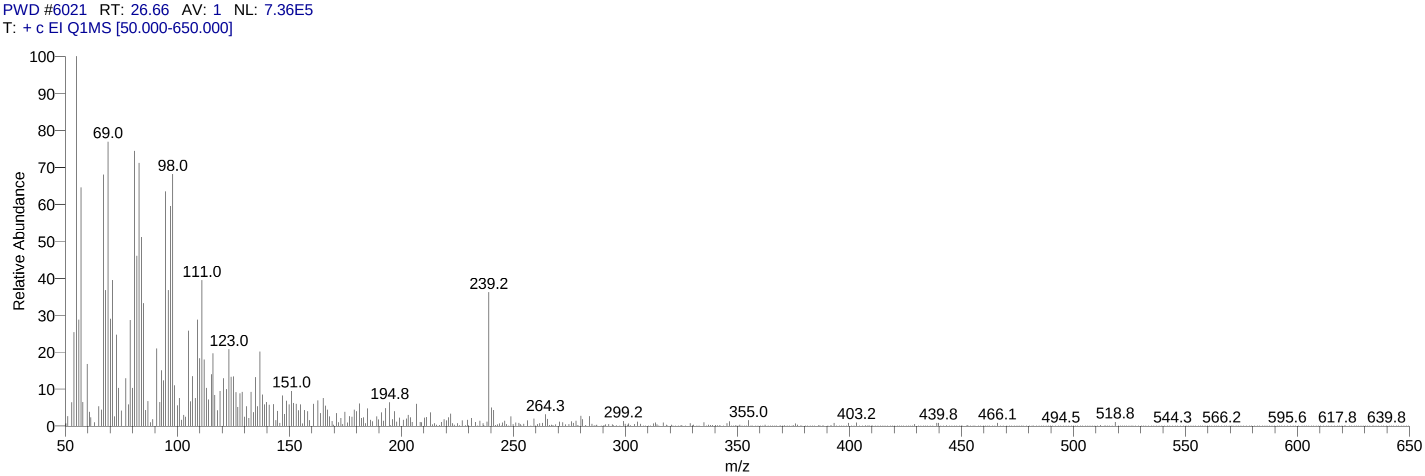

RT	Compound Name	Area %	Molecular Formula	Molecular Weight	Cas #	MF	Library
26.66	9-OCTADECENOIC ACID (Z)-	0.91	C18H34O2	282	112-80-1	797	WileyRegistry8e
26.66	trans-13-Octadecenoic acid	0.91	C18H34O2	282	693-71-0	784	mainlib
26.66	12-Methyl-E,E-2,13-octadecadien-1-ol	0.91	C19H36O	280	NA	816	mainlib

My GC-MS Report

RT	Compound Name	Area %	Molecular Formula	Molecular Weight	Cas #	MF	Library
26.66	cis-Vaccenic acid	0.91	C18H34O2	282	506-17-2	781	mainlib
26.66	9-Octadecenoic acid, (E)-	0.91	C18H34O2	282	112-79-8	805	replib

Compound Structure | Hit Spectrum

9-OCTADECENOIC ACID (Z)-
Formula C18H34O2, MW 282, CAS# 112-80-1, Entry# 172910
OCTADEC-9-ENOIC ACID

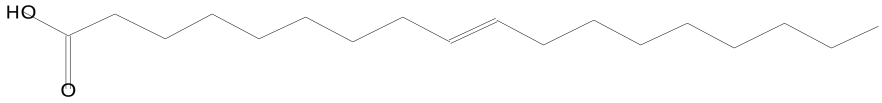

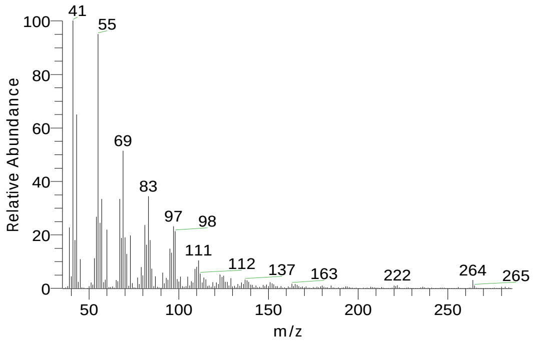

trans-13-Octadecenoic acid
Formula C18H34O2, MW 282, CAS# 693-71-0, Entry# 19306
$:28BDLLSHRIFPDGQB-AATRIKPKSA-N

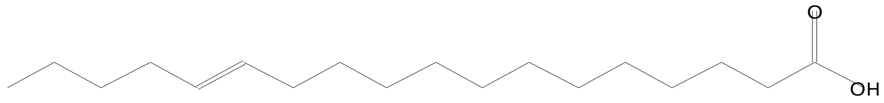

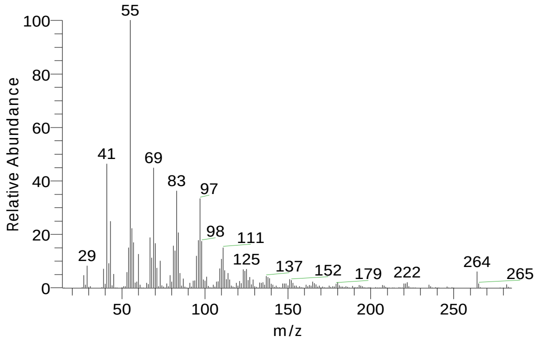

12-Methyl-E,E-2,13-octadecadien-1-ol
Formula C19H36O, MW 280, CAS# NA, Entry# 19016
(2E,15Z)-14-Methyl-2,15-octadecadien-1-ol #

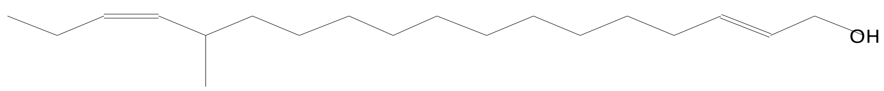

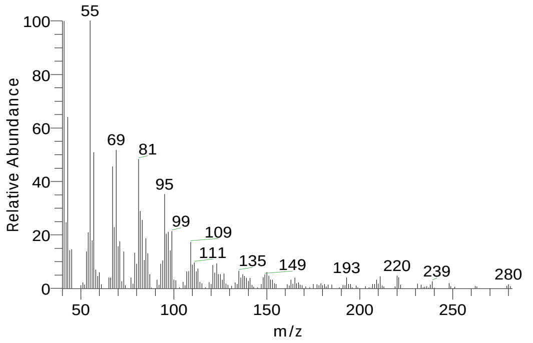

cis-Vaccenic acid
Formula C18H34O2, MW 282, CAS# 506-17-2, Entry# 20090
11-Octadecenoic acid, (Z)-

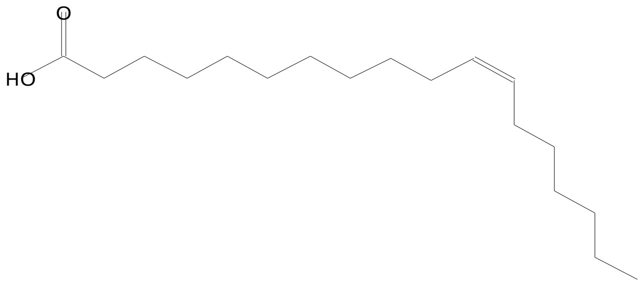

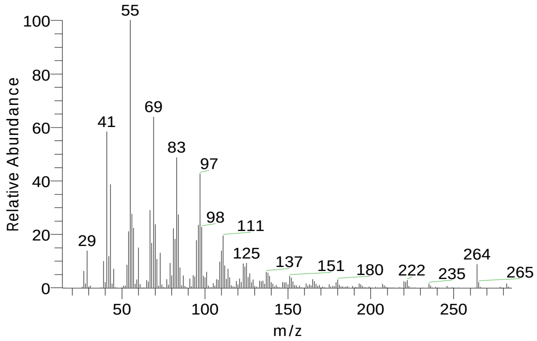

My GC-MS Report

Compound Structure

9-Octadecenoic acid, (E)-
Formula C18H34O2, MW 282, CAS# 112-79-8, Entry# 5015
trans-ë(sup 9)-Octadecenoic acid

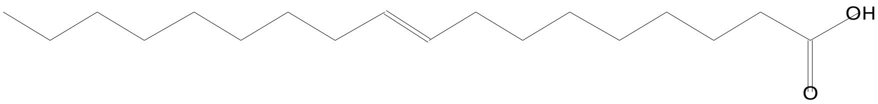

Hit Spectrum

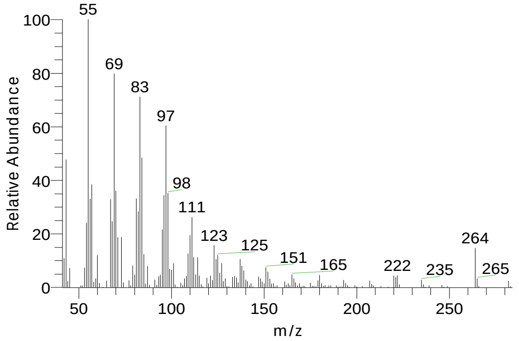

PWD #6636 RT: 28.98 AV: 1 NL: 8.84E5
T: + c EI Q1MS [50.000-650.000]

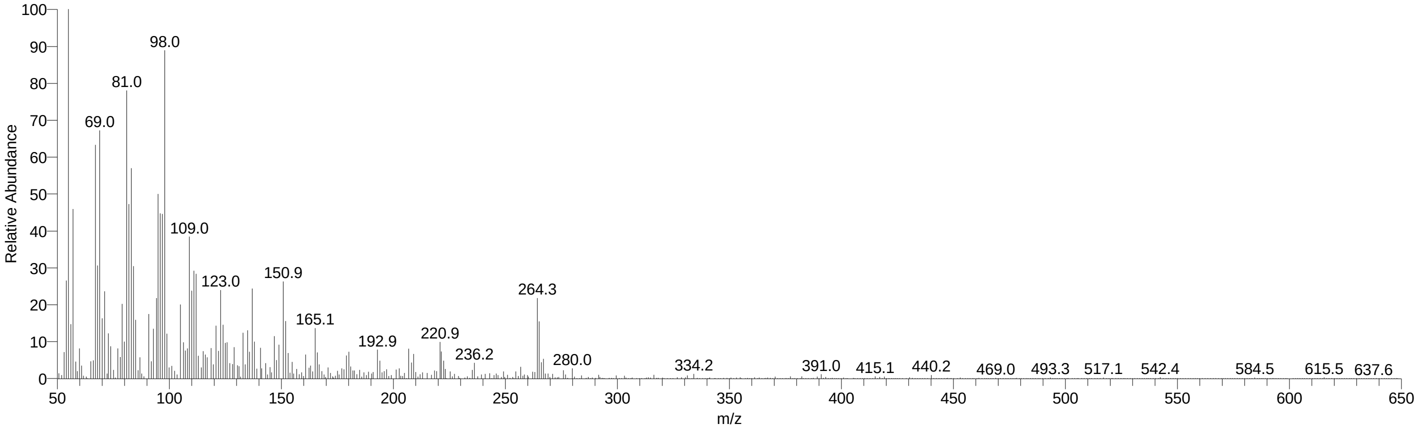

RT	Compound Name	Area %	Molecular Formula	Molecular Weight	Cas #	MF	Library
28.98	9-OCTADECENOIC ACID (Z)-, 2-HYDROXY-1-(HYDROXYMETHYL)ETHYL ESTER	3.60	C21H40O4	356	3443-84-3	825	WileyRegistry8e
28.98	9-Octadecenoic acid (Z)-, 2-hydroxy-1-(hydroxymethyl)ethyl ester	3.60	C21H40O4	356	3443-84-3	825	mainlib
28.98	9-Octadecenoic acid (Z)-, 2,3-dihydroxypropyl ester	3.60	C21H40O4	356	111-03-5	831	replib
28.98	9-OCTADECENOIC ACID (Z)-, 2,3-DIHYDROXYPROPYL ESTER	3.60	C21H40O4	356	111-03-5	830	WileyRegistry8e
28.98	Oleic anhydride	3.60	C36H66O3	546	24909-72-6	800	mainlib

Compound Structure

9-OCTADECENOIC ACID (Z)-, 2-HYDROXY-1-(HYDROXYMETHYL)ETHYL ESTER
Formula C21H40O4, MW 356, CAS# 3443-84-3, Entry# 232585
2-HYDROXY-1-(HYDROXYMETHYL)ETHYL (9Z)-9-OCTADECENOATE #

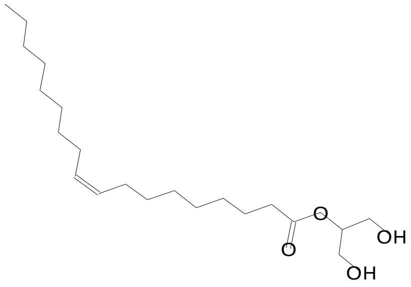

Hit Spectrum

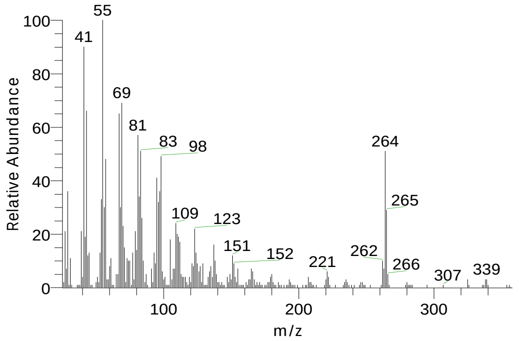

My GC-MS Report

Compound Structure | Hit Spectrum

9-Octadecenoic acid (Z)-, 2-hydroxy-1-(hydroxymethyl)ethyl ester
Formula C21H40O4, MW 356, CAS# 3443-84-3, Entry# 19276
Olein, 2-mono-

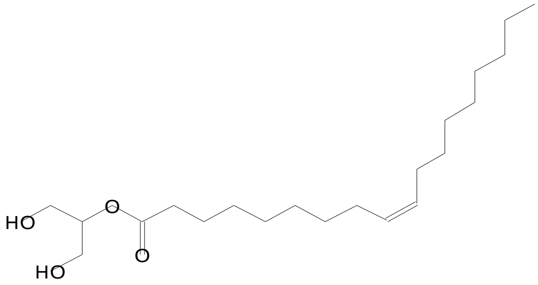

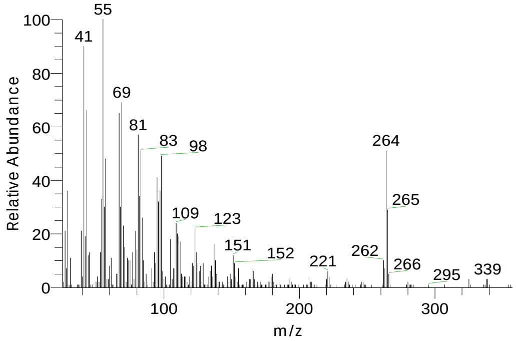

9-Octadecenoic acid (Z)-, 2,3-dihydroxypropyl ester
Formula C21H40O4, MW 356, CAS# 111-03-5, Entry# 4731
Glyceryl Monooleate

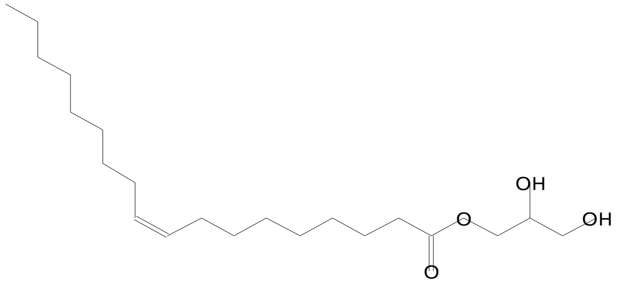

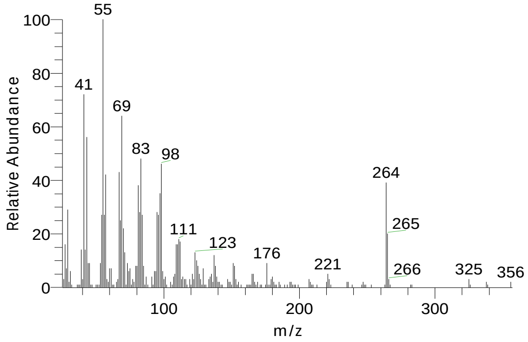

9-OCTADECENOIC ACID (Z)-, 2,3-DIHYDROXYPROPYL ESTER
Formula C21H40O4, MW 356, CAS# 111-03-5, Entry# 232582
2,3-DIHYDROXYPROPYL (9Z)-9-OCTADECENOATE #

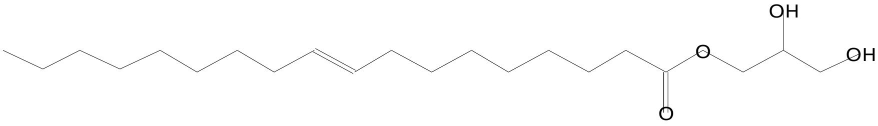

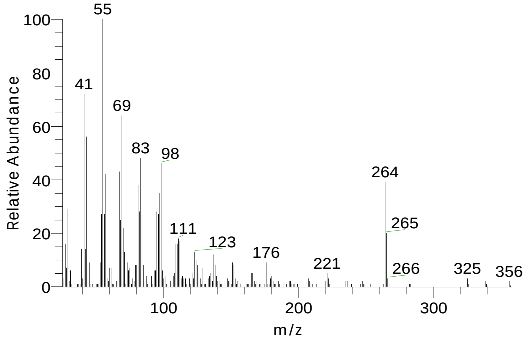

Oleic anhydride
Formula C36H66O3, MW 546, CAS# 24909-72-6, Entry# 70991
9-Octadecenoic acid (Z)-, anhydride

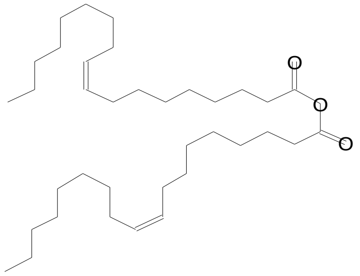

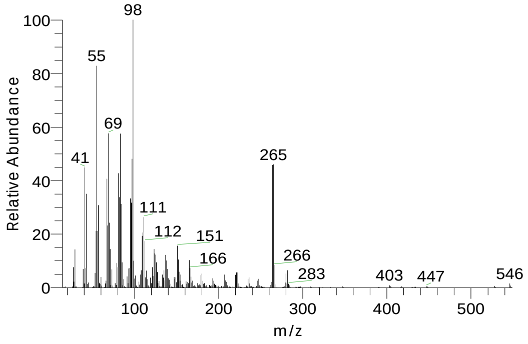

My GC-MS Report

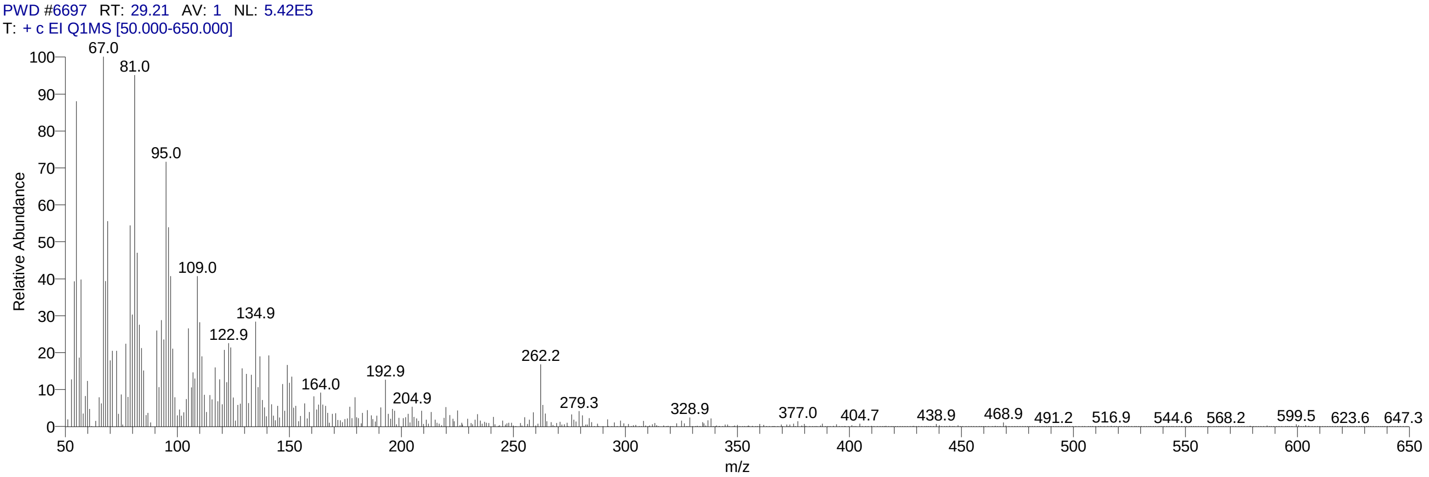

RT	Compound Name	Area %	Molecular Formula	Molecular Weight	Cas #	MF	Library
29.21	9,12-Octadecadienoic acid (Z,Z)-, 2-hydroxy-1-(hydroxymethyl)ethyl ester	1.08	C21H38O4	354	3443-82-1	808	replib
29.21	Linoleic acid ethyl ester	1.08	C20H36O2	308	544-35-4	782	replib
29.21	9,12-Octadecadienoic acid (Z,Z)-, 2,3-dihydroxypropyl ester	1.08	C21H38O4	354	2277-28-3	796	mainlib
29.21	9,12-OCTADECADIENOIC ACID (Z,Z)-, 2,3-DIHYDROXYPROPYL ESTER	1.08	C21H38O4	354	2277-28-3	791	WileyRegistry8e
29.21	E,E,Z-1,3,12-Nonadecatriene-5,14-diol	1.08	C19H34O2	294	NA	778	mainlib

Compound Structure	Hit Spectrum

9,12-Octadecadienoic acid (Z,Z)-, 2-hydroxy-1-(hydroxymethyl)ethyl ester
Formula C21H38O4, MW 354, CAS# 3443-82-1, Entry# 8055
Linolein, 2-mono-

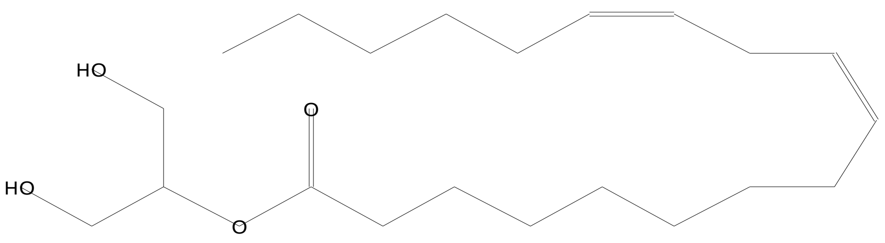

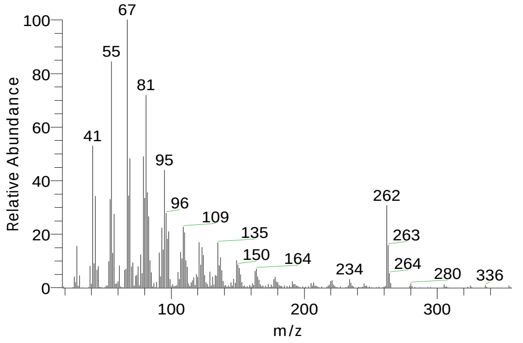

Linoleic acid ethyl ester
Formula C20H36O2, MW 308, CAS# 544-35-4, Entry# 8097
Ethyl linoleate

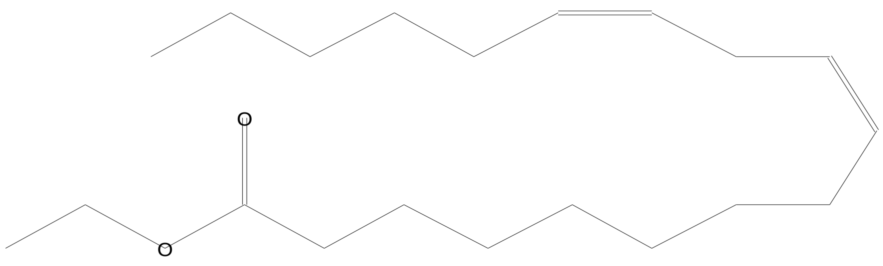

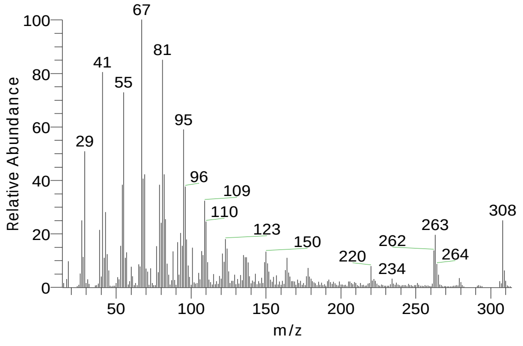

My GC-MS Report

Compound Structure	Hit Spectrum

9,12-Octadecadienoic acid (Z,Z)-, 2,3-dihydroxypropyl ester
Formula C21H38O4, MW 354, CAS# 2277-28-3, Entry# 32821
Linolein, 1-mono-

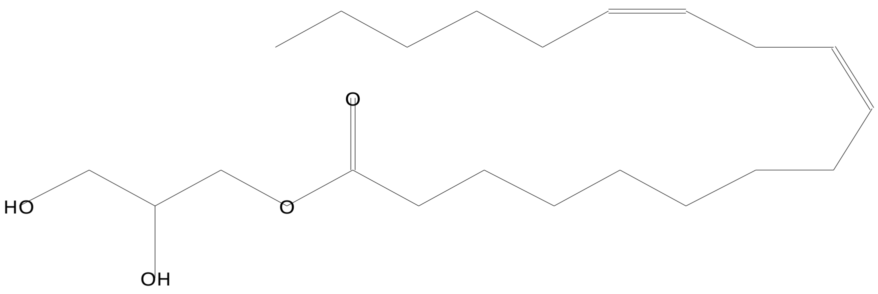

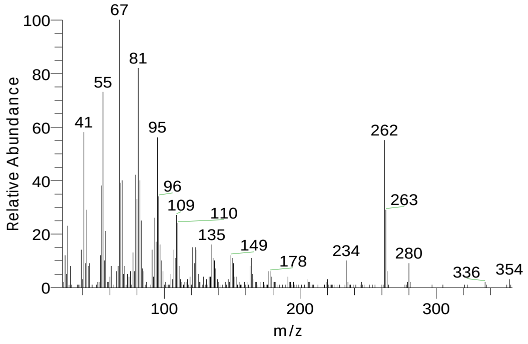

9,12-OCTADECADIENOIC ACID (Z,Z)-, 2,3-DIHYDROXYPROPYL ESTER
Formula C21H38O4, MW 354, CAS# 2277-28-3, Entry# 231385
2,3-DIHYDROXYPROPYL (9Z,12Z)-9,12-OCTADECADIENOATE #

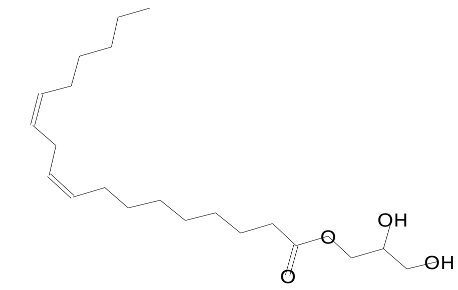

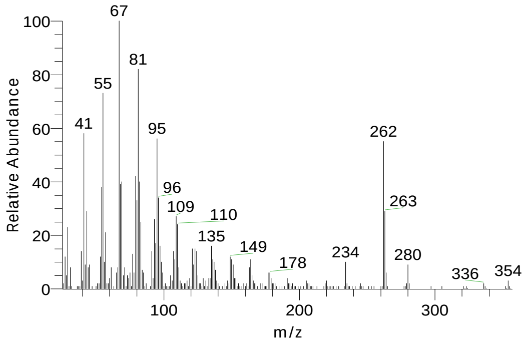

E,E,Z-1,3,12-Nonadecatriene-5,14-diol
Formula C19H34O2, MW 294, CAS# NA, Entry# 21026
(3E,12Z)-1,3,12-Nonadecatriene-5,14-diol #

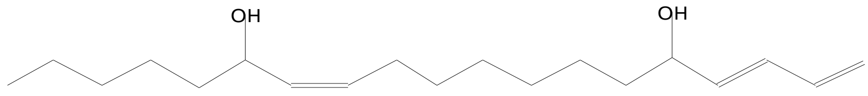

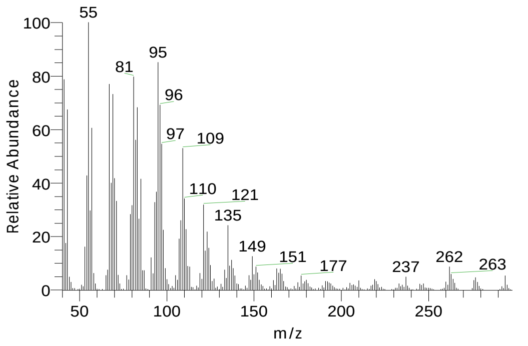

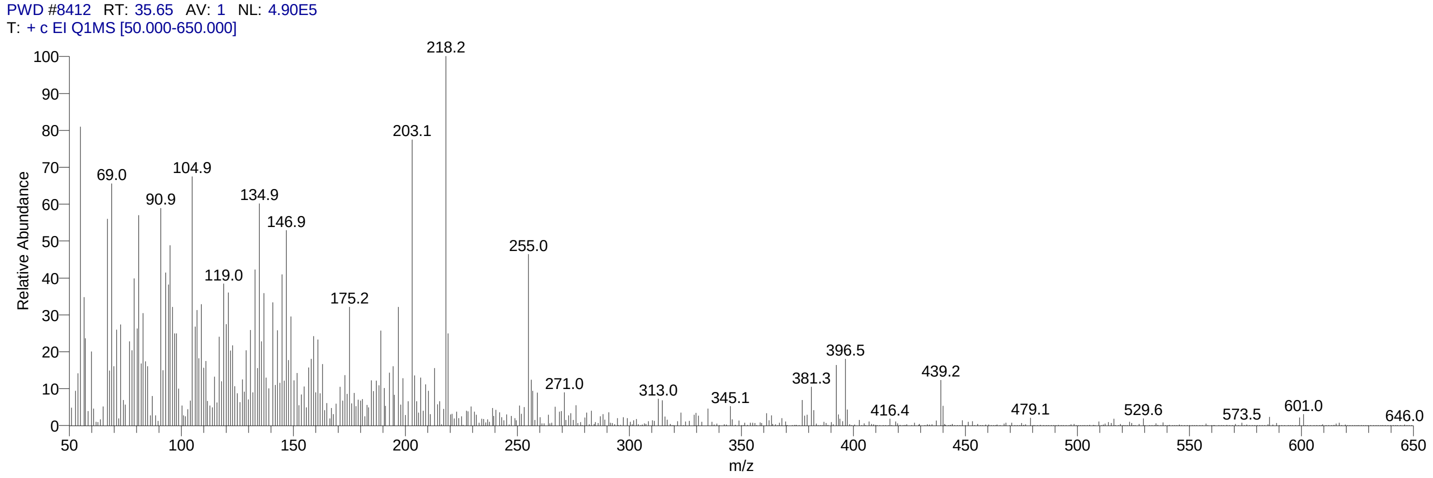

RT	Compound Name	Area %	Molecular Formula	Molecular Weight	Cas #	MF	Library
35.65	STIGMAST-5-EN-3-OL, (3á,24S)-	3.08	C29H50O	414	83-47-6	725	WileyRegistry8e
35.65	METHYL COMMATE C	3.08	C31H50O4	486	NA	763	WileyRegistry8e

My GC-MS Report

RT	Compound Name	Area %	Molecular Formula	Molecular Weight	Cas #	MF	Library
35.65	METHYL COMMATE D	3.08	C31H50O4	486	NA	726	WileyRegistry8e
35.65	2-[4-methyl-6-(2,6,6-trimethylcyclohex-1-enyl)hexa-1,3,5-trienyl]cyclohex-1-en-1-carboxaldehyde	3.08	C23H32O	324	NA	740	mainlib
35.65	STIGMAST-5-EN-3-OL, (3á)-	3.08	C29H50O	414	83-46-5	703	WileyRegistry8e

Compound Structure	Hit Spectrum

STIGMAST-5-EN-3-OL, (3á,24S)-
Formula C29H50O, MW 414, CAS# 83-47-6, Entry# 262359
STIGMAST-5-EN-3-OL #

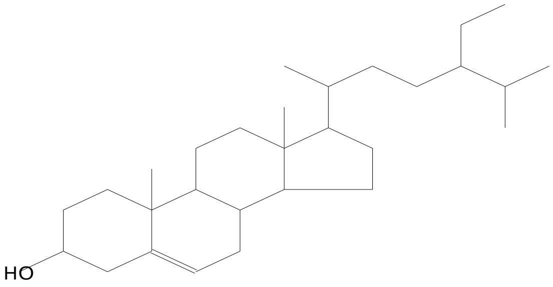

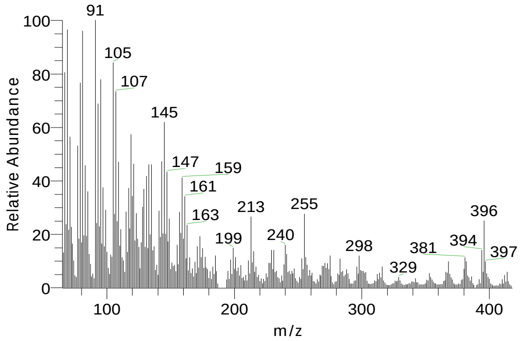

METHYL COMMATE C
Formula C31H50O4, MW 486, CAS# NA, Entry# 283159

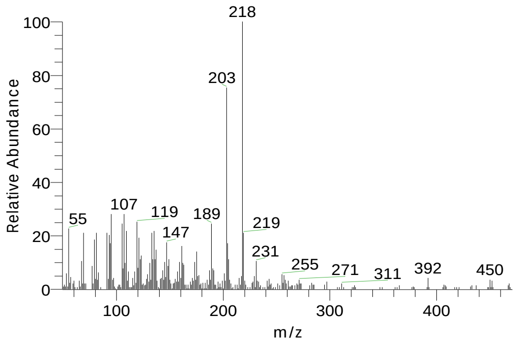

METHYL COMMATE D
Formula C31H50O4, MW 486, CAS# NA, Entry# 283158

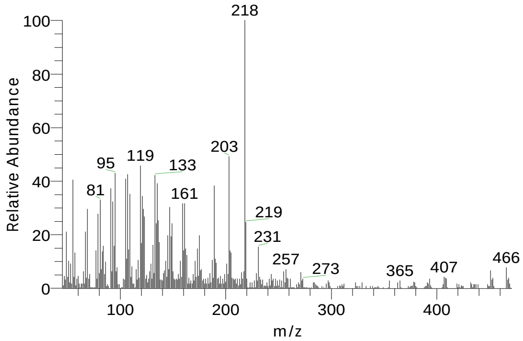

Formula C23H32O, MW 324, CAS# NA, Entry# 5913

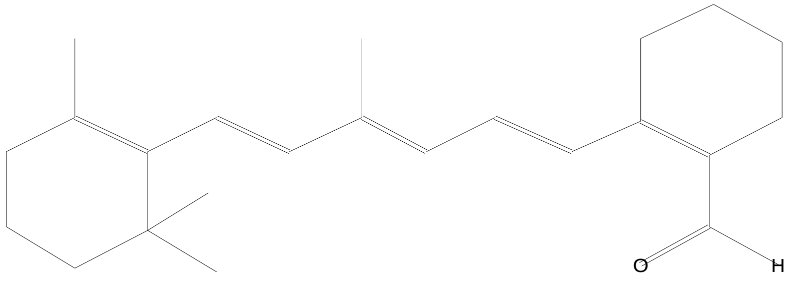

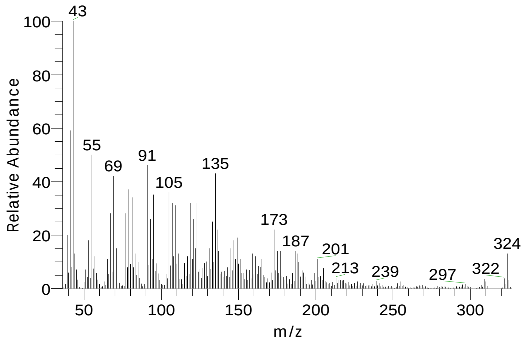

My GC-MS Report

Compound Structure | Hit Spectrum

STIGMAST-5-EN-3-OL, (3á)-
Formula C29H50O, MW 414, CAS# 83-46-5, Entry# 262352

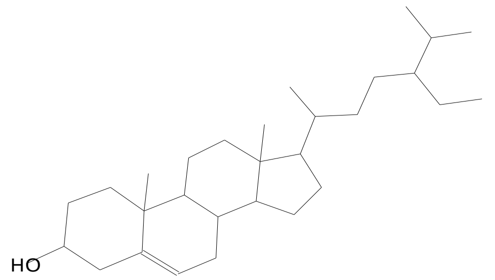

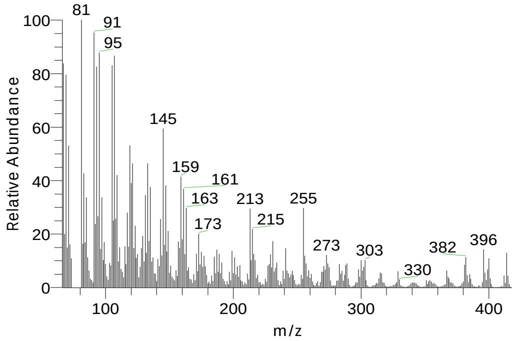

PWD #8565 RT: 36.23 AV: 1 NL: 1.52E6
T: + c EI Q1MS [50.000-650.000]

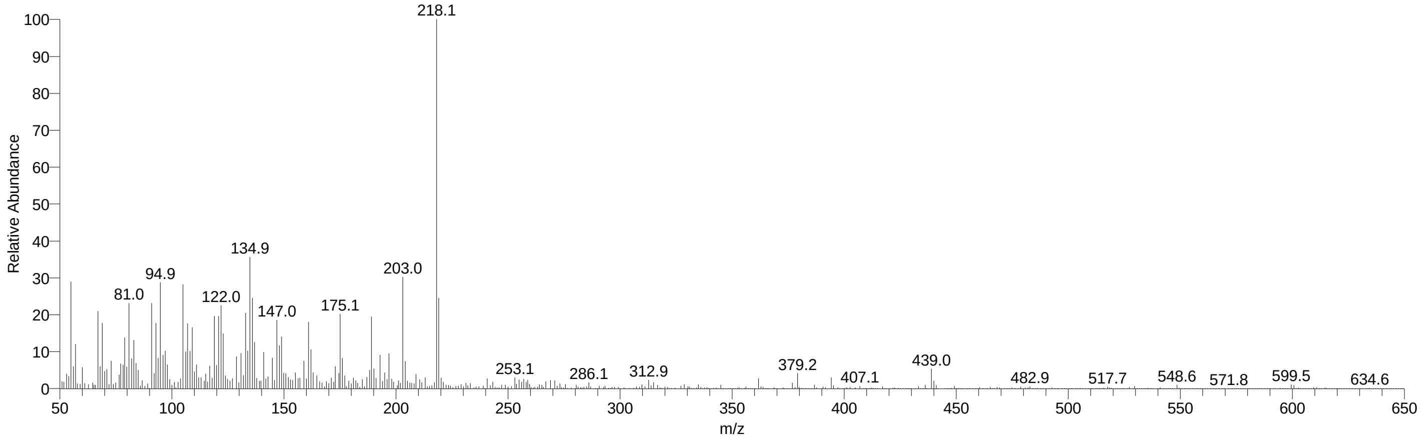

RT	Compound Name	Area %	Molecular Formula	Molecular Weight	Cas #	MF	Library
36.23	24-Norursa-3,12-diene	3.45	C29H46	394	201358-25-0	802	mainlib
36.23	24-Noroleana-3,12-diene	3.45	C29H46	394	201358-24-9	797	mainlib
36.23	METHYL COMMATE C	3.45	C31H50O4	486	NA	786	WileyRegistry8e
36.23	METHYL COMMATE B	3.45	C31H50O3	470	NA	777	WileyRegistry8e
36.23	METHYL COMMATE D	3.45	C31H50O4	486	NA	756	WileyRegistry8e

Compound Structure | Hit Spectrum

24-Norursa-3,12-diene
Formula C29H46, MW 394, CAS# 201358-25-0, Entry# 194805

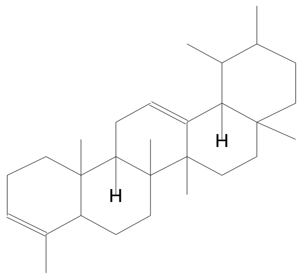

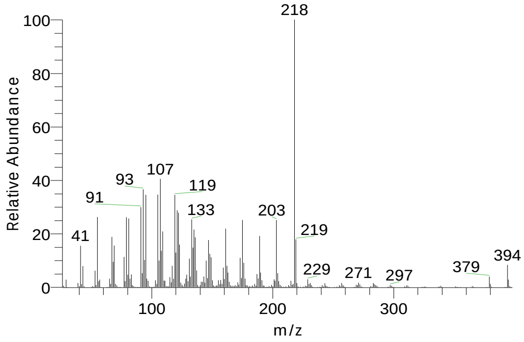

My GC-MS Report

Compound Structure | Hit Spectrum

24-Noroleana-3,12-diene
Formula C29H46, MW 394, CAS# 201358-24-9, Entry# 195020

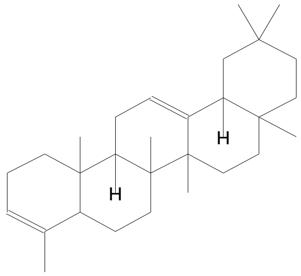

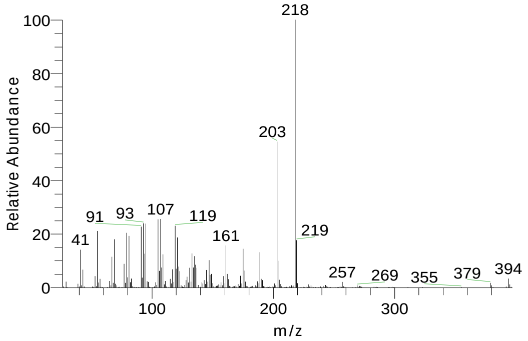

METHYL COMMATE C
Formula C31H50O4, MW 486, CAS# NA, Entry# 283159

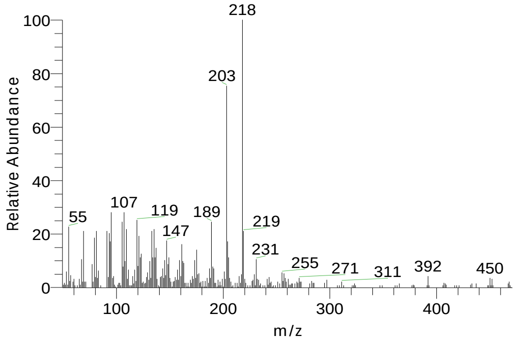

METHYL COMMATE B
Formula C31H50O3, MW 470, CAS# NA, Entry# 279570

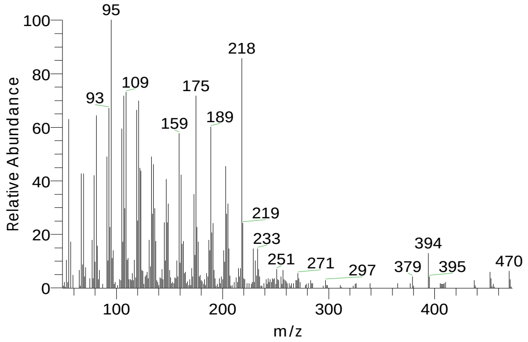

METHYL COMMATE D
Formula C31H50O4, MW 486, CAS# NA, Entry# 283158

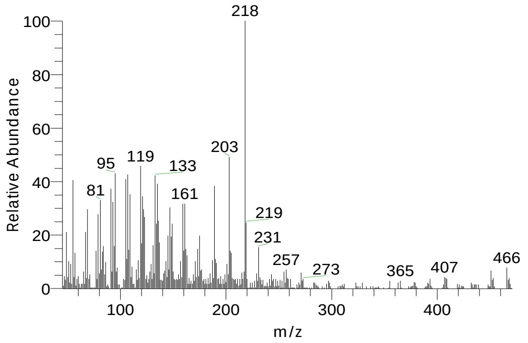

My GC-MS Report

PWD #9701 RT: 40.49 AV: 1 NL: 4.59E5
T: + c EI Q1MS [50.000-650.000]

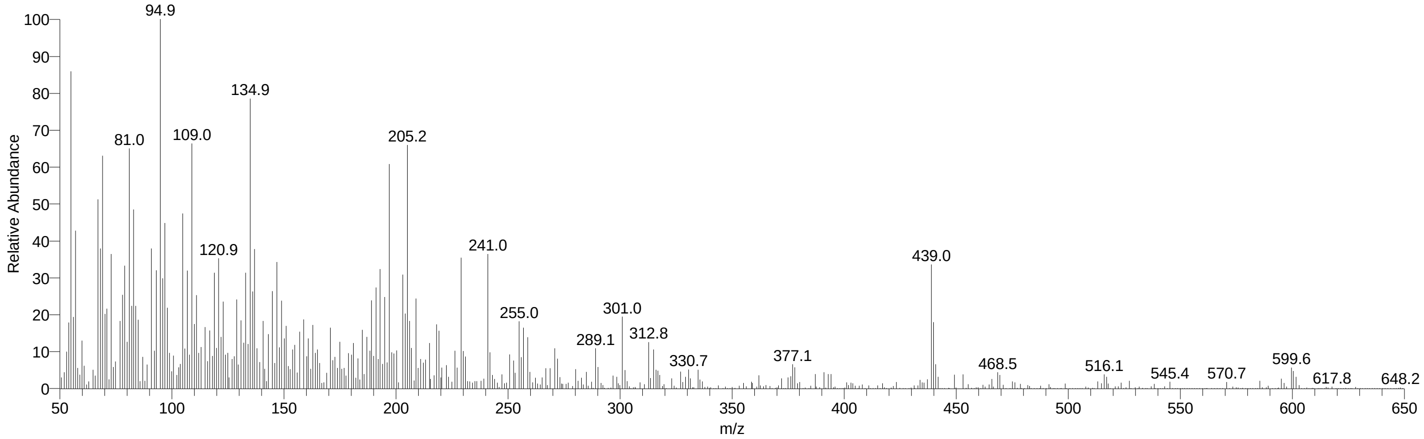

RT	Compound Name	Area %	Molecular Formula	Molecular Weight	Cas #	MF	Library
40.49	D:A-Friedo-2,3-secooleanane-2,3-dioic acid, dimethyl ester, (4R)-	2.35	C32H54O4	502	88373-58-4	649	mainlib
40.49	METHYL 2-[1-(2-METHOXY-2-OXOETHYL)-2,4B,6A,9,9,10B,12A-HEPTAMETHYLOCTADECAHYDRO-2-CHRYSENYL]PROPANOATE #	2.35	C32H54O4	502	88373-58-4	649	WileyRegistry8e
40.49	9,19-Cyclochloestene-3,7-diol, 4,14-dimethyl-, 3-acetate	2.35	C31H52O3	472	NA	651	mainlib
40.49	<No Name>	2.35	C31H52O3	472	NA	651	WileyRegistry8e
40.49	Betulin	2.35	C30H50O2	442	473-98-3	644	replib

Compound Structure | Hit Spectrum

D:A-Friedo-2,3-secooleanane-2,3-dioic acid, dimethyl ester, (4R)-
Formula C32H54O4, MW 502, CAS# 88373-58-4, Entry# 68094

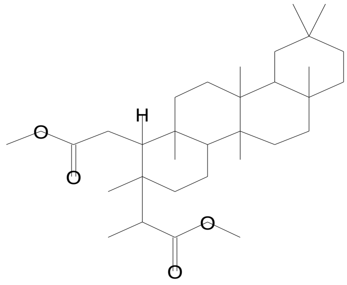

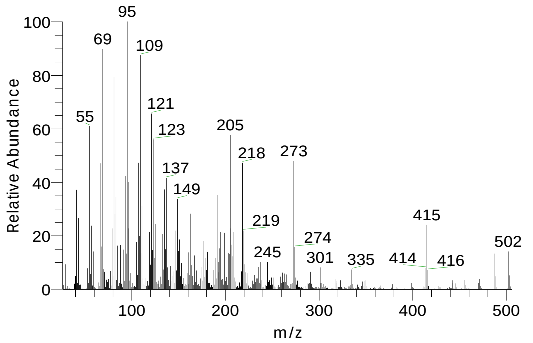

Formula C32H54O4, MW 502, CAS# 88373-58-4, Entry# 374089
D:A-FRIEDO-2,3-SECOOLEANANE-2,3-DIOIC ACID, DIMETHYL ESTER, (4R)-

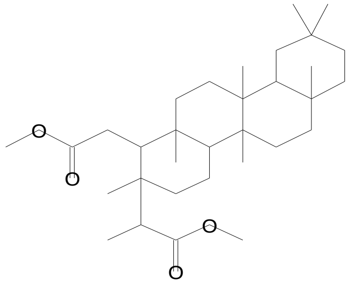

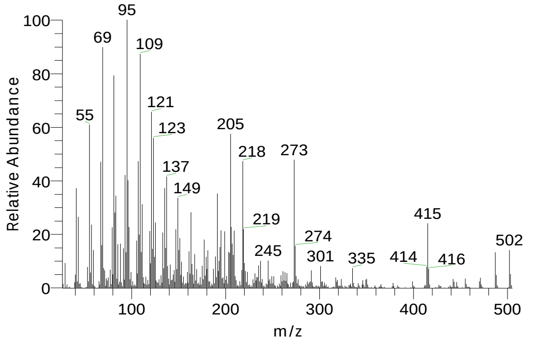

My GC-MS Report

Compound Structure | Hit Spectrum

9,19-Cyclochloestene-3,7-diol, 4,14-dimethyl-, 3-acetate
Formula C31H52O3, MW 472, CAS# NA, Entry# 174414

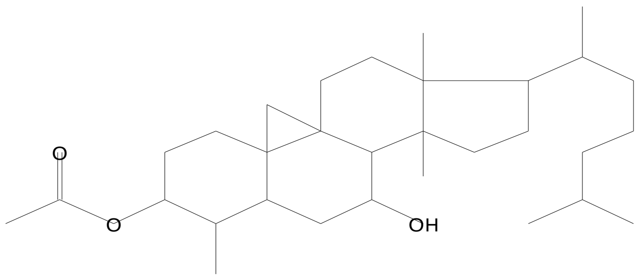

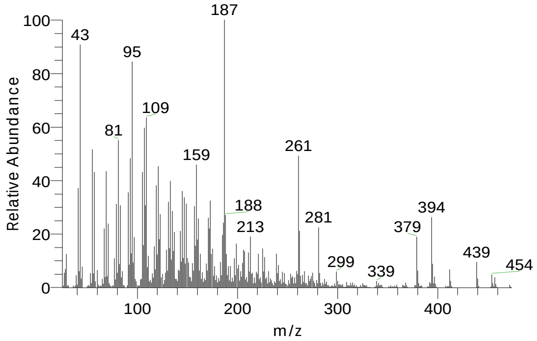

<No Name>
Formula C31H52O3, MW 472, CAS# NA, Entry# 375102

SI 611, RSI 651, WileyRegistry8e, Entry# 375102, CAS# NA, <No Name>

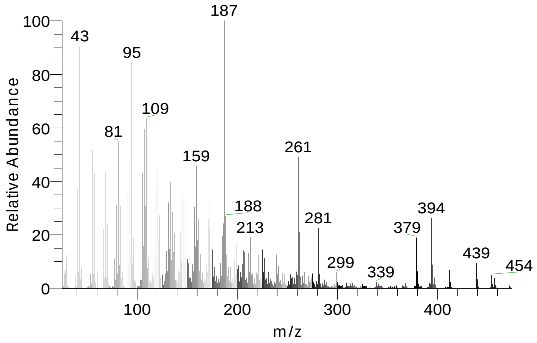

Betulin
Formula C30H50O2, MW 442, CAS# 473-98-3, Entry# 27738
Lup-20(29)-ene-3,28-diol, (3á)-

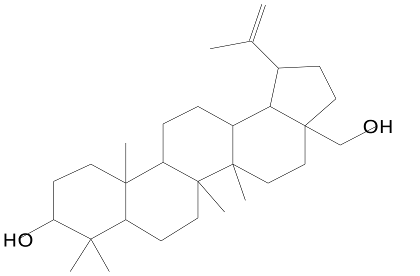

SI 598, RSI 644, replib, Entry# 27738, CAS# 473-98-3, Betulin

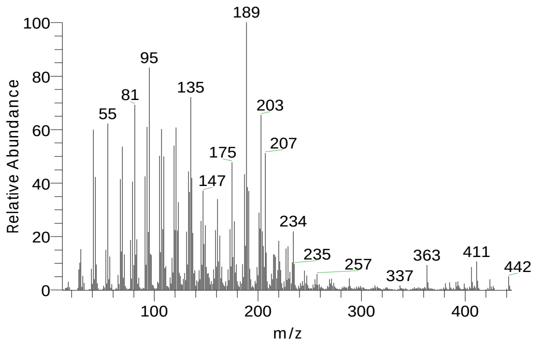

Supplement: Supplementary file 1 [file DataSheet1.pdf]
